# *In silico *comparative analysis of SSR markers in plants

**DOI:** 10.1186/1471-2229-11-15

**Published:** 2011-01-19

**Authors:** Filipe C Victoria, Luciano C da Maia, Antonio Costa de Oliveira

**Affiliations:** 1 Plant Genomics and Breeding Center, Faculdade de Agronomia Eliseu Maciel, Universidade Federal de Pelotas, RS, Brasil; 2 Graduate Program in Biotechnology, Universidade Federal de Pelotas, RS, Brasil

## Abstract

**Background:**

The adverse environmental conditions impose extreme limitation to growth and plant development, restricting the genetic potential and reflecting on plant yield losses. The progress obtained by classic plant breeding methods aiming at increasing abiotic stress tolerances have not been enough to cope with increasing food demands. New target genes need to be identified to reach this goal, which requires extensive studies of the related biological mechanisms. Comparative analyses in ancestral plant groups can help to elucidate yet unclear biological processes.

**Results:**

In this study, we surveyed the occurrence patterns of expressed sequence tag-derived microsatellite markers for model plants. A total of 13,133 SSR markers were discovered using the *SSRLocator *software in non-redundant EST databases made for all eleven species chosen for this study. The dimer motifs are more frequent in lower plant species, such as green algae and mosses, and the trimer motifs are more frequent for the majority of higher plant groups, such as monocots and dicots. With this *in silico *study we confirm several microsatellite plant survey results made with available bioinformatics tools.

**Conclusions:**

The comparative studies of EST-SSR markers among all plant lineages is well suited for plant evolution studies as well as for future studies of transferability of molecular markers.

## Background

In agriculture, productivity is affected by environmental conditions such as drought, salinity, high radiation and extreme temperatures faced by plants during their life cycle, that impose severe limitations to the growth and propagation, restricting their genetic potential and, ultimately, reflecting yield losses of agricultural crops. Although, advances have been achieved through classical breeding, further progress is needed to increase abiotic stress tolerance in cultivated plants. New gene targets need to be identified in order to reach these goals, requiring extensive studies concerning the biological processes related to abiotic stresses. Comparative analysis between primitive and related groups of cultivated species may shed some light on the understanding of these processes.

Microsatellites or SSRs (Simple Sequence Repeats) are sequences in which one or few bases are tandemly repeated, ranging from 1-6 base pair (bp) long units. They are ubiquitous in prokaryotes and eukaryotes, present even in the smallest bacterial genomes [1-3]. Variations in SSR regions originate mostly from errors during the replication process, frequently DNA Polymerase slippage. These errors generate base pair insertions or deletions, resulting, respectively, in larger or smaller regions [4]. SSR assessments in the human genome have shown that many diseases are caused by mutation in these sequences [5]. The genomic abundance of microsatellites, and their ability to associate with many phenotypes, make this class of molecular markers a powerful tool for diverse application in plant genetics. The identification of microsatellite markers derived from EST (or cDNAs), and described as functional markers, represents an even more useful possibility for these markers when compared to those based on assessing anonymous regions [6-8]. EST-SSRs offer some advantages over other genomic DNA-based markers, such as detecting the variation in the expressed portion of the genome, giving a ''perfect''marker-trait association; they can be developed from EST databases at no cost and unlike genomic SSRs, they may be used across a number of related species [9].

Many studies indicate UTRs as being more abundant in microsatellites than CDS regions [10]. In a study of micro- and minisatellite distribution in UTR and CDS regions using the Unigene database for several higher plants groups, higher occurrence of these elements in coding regions were found for all the studied species [11]. Disagreements between earlier reports and the later, reflect a deficiency in annotation when translated and non-translated fractions are separated in the Unigene transcript database. Dimer repeats were also frequent in CDS regions, which could be due to the fact that the Unigene database contains predominantly EST clusters. Therefore, there is a tendency for under-representing the UTR regions in the annotated sequences [11].

The characterization of tandem repeats and their variation within and between different plant families, could facilitate their use as genetic markers and consequently allow plant-breeding strategies that focus on the transfer of markers from model to orphan species to be applied. EST-SSR also have a higher probability of being in linkage disequilibrium with genes/QTLs controlling economic traits, making them more useful in studies involving marker-trait association, QTL mapping and genetic diversity analysis [9].

On model organisms, microsatellites have been reported to correspond to 0.85% of *Arabidopsis thaliana *(L.) Heynh, 0.37% of maize (*Zea mays *L.), 3.21% of tiger puffer (*Takifugu rubripes *Temminck & Schlegel), 0.21% of the nematode *Caenorhabditis elegans *Maupas and 0.30% of yeast (*Saccharomyces cerevisiae *Meyer ex. E.C. Hansen) genomes [10]. Moreover, they constitute 3.00% of the human genome [12]. All kinds of repeated element motifs, excluding trimers and hexamers, are significantly less frequent in the coding sequences when compared to intergenic DNA streches of *A. thaliana*, *Z. mays, Oryza sativa *subsp *japonica *S. Kato (rice), *Glycine max *(L.) Merr. (soybean) and *Triticum aestivum *L. (wheat) [10].

Close to 48.67% of repeat elements found in many species are formed by dimer motifs. In *Picea abies *(L.) H. Karst. (Norway spruce), for example, the dimer occurrence is 20 times more frequent in clones originating from intergenic regions vs. transcript regions [13]. Approximately 14% of protein translated sequences (CDS - coding sequences) contain repetitive DNA regions, and this phenomenon is 3 folds more frequent in eukaryotes than prokaryotes [14]. Clustering studies showing microsatellite occurrence in distinct protein families (non-homologous) from either prokaryotic or eukaryotic genomes, indicate that the origins of these loci occurred after eukaryotic evolution [14-16]. The highest and lowest repeat counts were found in rodents and *C. elegans*, respectively [3].

In plant species, some reports have described the levels of occurrence of microsatellites associated to transcribed regions [7,8,10,11,17-22]. However, some comparative and/or descriptive approaches, still can offer new perspectives on the features of these markers. Furthermore, frequently new groups of plant species have their genome sequenced, enabling the reassessment of databases using new sequences, representing divergent evolutionary groups and/or with different genetic models.

The online platforms for nucleotide, protein and transcript (ESTs) databases available for the majority of species are relatively small when compared with model species, eg *Physcomitrella patens *(Hedw.) Bruch & Schimp., *O. sativa *and *A. thaliana*. Since the protocols for the isolation of repetitive element loci, such as microsatellites, require intensive labour and can be expensive, the exploitation of these elements *in silico *on databases of model plants and their respective transfer to orphan species, is a potentially fruitful strategy.

In this study we present our results on the SSR survey for the development of plant SSR markers. The survey was based on clustered non-redundant EST data, their classification, characterization and comparative analysis in eleven phylogenetically distant plant species including two green algae, a hepatic, two mosses, two fern, two gymnosperms, a monocot and a dicot.

## Results and Discussion

We analysed 560,360 virtual transcripts with the *SSRLocator *software (Table 1). The species with most abundant records in Genbank was *Arabidopsis thaliana *with 224,496 virtual transcripts (40%), followed by *Oryza sativa *with 121,635 (21.7%), *Physcomitrela patens *with 79,537 (14.19%), *Pinus taeda *with 58,522 (10.44%) and *Chlamydomonas reinhardtii *with 40,525 (7.2%). The remaining species added up to 11.7% of virtual transcripts analysed. When total genome sizes are compared for the model plants included in this analysis, the virtual transcripts of *P. patens *(511 Mb) represent 0.01% of genome size. For *O. sativa *(389 Mb) and *A. thaliana *(109.2 Mb) the ESTs analysed represent 0.02% and 0.18%, respectively, of the genome. The highest average bp count per EST sequence was found for *Selaginella *spp. (924 bp) followed by *M. polymorpha *(777 bp), *C. reinhardtii *(775 bp) and *P. taeda *(760 bp). The lower average bp per sequence was found for *G. gnemon *(563 bp) and *A. capillus-veneris *(580 bp). For the model plants, *A. thaliana *showed the lowest average bp count (321 bp), with *P. patens *and *O. sativa *presenting similar bp counts (737 and 755 bp, respectively). Shorter observed sequences could be an indication of incomplete representation of genes, but one must keep in mind that average gene sizes could vary among species, i.e., rice fl-cDNAs (1,747 bp) are 14% longer than *Arabidopsis *fl-cDNAs (1,532 bp) (TAIR 9 and RIKEN, accessed in 12.2.2010). The overall bp counts are very similar to those found by other authors [23].

**Table 1:** EST database size and Overall occurrence of SSR, percentages and average length motifs per specie

***Species***	***EST database count***	***pb***	***Average pg count per EST***	***GC Content %***
*Chlamydomonas reinhardtii*	40,525	31,388,333	775	57.22
*Mesostigma viride*	6,401	4,273,634	668	51.36
*Marchantia polymorpha*	10,086	7,836,025	777	54.75
*Syntrichia ruralis*	7,114	4,764,692	670	49.20
*Physcomitrella patens*	79,537	58,636,814	737	47.60
*Selaginella spp.*	19,830	18,318,250	924	51.38
*Adiantum capillus-veneris*	16,138	9,363,530	580	45.97
*Gnetum gnemon*	6,076	3,420,021	563	44.33
*Pinus taeda*	58,522	44,467,932	760	43.64
*Oryza sativa*	121,635	91,859,132	755	47.52
*Arabidopsis thaliana*	224,496	72,013,660	321	41.10

The frequency of SSR per EST database was higher (4.66%) in *Selaginella *spp virtual transcripts (Table 2). For model plants, 3.57% and 0.84% SSRs/EST were found for *O. sativa *and *A. thaliana*, respectively.

**Table 2:** EST database size and Overall occurrences of SSRs, percentages and average length motifs per species

***Species***	***Number of SSR loci***	***SSR/EST database (%)***	***Average motif length (bp)***	***EST sequences with SSRs ******(%)***	***N. of seq. containing more than one SSR ******(%)***	***Single SSRs***	***Compound SSRs***
*Chlamydomonas reinhardtii*	980	2.41	33.21	886 (2.19)	94 (9.78)	899	81
*Mesostigma viride*	81	1.26	34.12	73 (1.14)	8 (9.87)	73	8
*Marchantia polymorpha*	437	4.33	22.56	436 (4.32)	1 (0.52)	425	12
*Syntrichia ruralis*	190	2.67	23.84	149 (2.09)	41 (10.09)	189	1
*Physcomitrella patens*	2753	3.46	24.20	2577 (3.24)	176 (6.6)	2670	83
*Selaginella spp.*	968	4.66	23.71	868 (4.38)	100 (11.13)	927	41
*Adiantum capillus-veneris*	749	4.64	31.14	599 (3.71)	150 (20.86)	624	125
*Gnetum gnemon*	212	3.48	23.62	195 (3.21)	17 (8.45)	203	9
*Pinus taeda*	568	0.97	30.89	530 (0.91)	38 (6.85)	539	29
*Oryza sativa*	4347	3.57	23.44	3934 (3.23)	413 (10.19)	4199	148
*Arabidopsis thaliana*	1890	0.84	26.52	1822 (0.81)	68 (3.62)	1837	53

The average motif length, excluding compound SSRs, was 27.03 bp. *Mesostigma *EST database shows the longest SSR average size with 34.13 bp, and the shortest size was found for *Marchantia polymorpha *with 22.56 bp mean size. The SSR size for model plants was similar. For *P. patens*, *O. sativa *and *A. thaliana*, average sizes of 24.2, 23.4 and 26.5 bp were found, respectively. A total 1,106 EST sequences contained more than one SSR. Among the species, *O. sativa *and *P. patens *are on the extremes of the distribution with 37.34% and 3.46% of virtual transcripts containing one or more microsatellites. However, *Adiantum capillus-veneris *EST database contained the highest percentage of transcripts displaying more than one SSR (20.86%) based on the database size. Similar results were found in our group [11], using the Unigene database for grasses and other allies. In the same study, rice was shown to have the highest frequency of ESTs containing more than one SSR (11.28%). In the present study, a similar value was found for rice (10.20%). These small differences could be due to different redundancy reduction parameters used in Unigene species database and CAP3 default settings. Other reports for higher plants [19,20,24-26], showed different ranges, but never higher than 2-3 fold. The variations encountered in different reports are related to the strategy employed by investigators (software, repeat number and motif type) [11]. The results for each species, regarding the percentage of SSRs found per EST database size are shown on Table 2.

The microsatellite survey using *SSRLocator *showed that 13,133 SSRs were available as potential marker loci. From those, 12,585 loci were found in single formation and only 590 were found in compound formation. The fern *A. capillus-veneris *showed the highest percentage (20%) of compound SSR loci. When compared with other available SSR marker search tools, similar results were found. Using MISA software, a total of 13,861 SSRs were available as potential marker loci, being 13,172 SSRs single and 689 compound SSRs for all studied species. *Adiantum *EST database showed the highest percentage of SSR in compound formation (15.55%). This trend does not hold for the majority of lower plants. *P. patens*, for example, presented few EST-SSRs in compound formation (3.57%) and possibly the fern lower database size is masking the results. When it is compared with the majority of plant groups, *P. taeda *is the only species showing a high percentage of compound SSRs (5.81%), corroborating other studies which report that compound and imperfect tandem repeats are most common in pines [27-29].

A total of 3,723 EST-SSRs were found in *P. patens *database using the MISA software [23]. The *SSRLocator *analysis resulted in 2,839 SSR for this species. When the same non-redundant databases were run in other bioformatics tools, the results were similar to MISA. Using the SciKoco package [30] combined with MISA, Sputinik and Modified scripts, it was possible to narrow SSR results to a 2-fold range variation.

**Figure 1:**
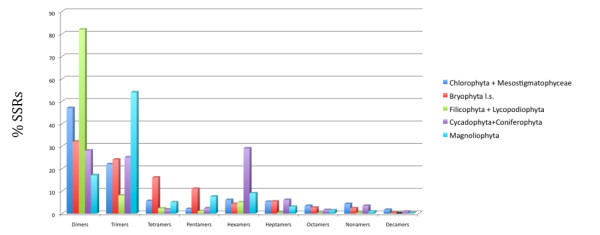
**SSR motifs occurrences by plant group studied**. SSR motifs (%) in all plant groups studied (Chlorophyta+Mesostigmatophyceae = unicelullar green algae; Bryophyta l.s. = hornworts, liverworts and mosses; Filicophyta+Lycopodiophyta = ferns; Cycadophyta+Coniferophyta = Gimnosperms; Magnoliophyta = flowering plants)

**Figure 2:**
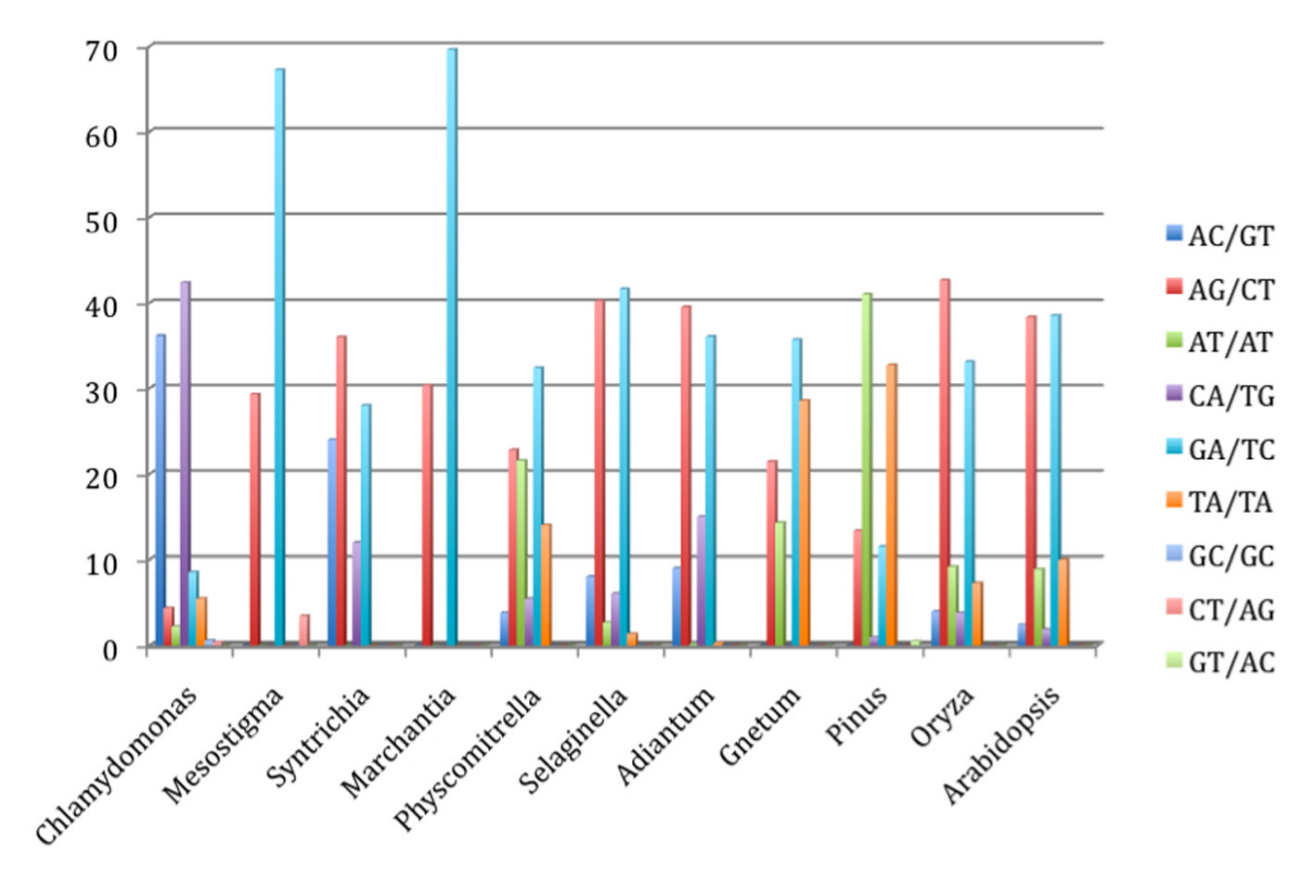
Predominant loci containing dinucleotide microsatellites motifs per species.

The search for repetitive elements in EST databases of the eleven taxa listed above enabled the comparison of patterns of occurrence of these elements in lower and higher plants (Figure 1). In some species such as *C. *reinhardtii, *Mesostigma viride *and bryophytes, we found that dimer (NN) microsatellites are more common when compared to higher plants (Figure 2). The trimer (NNN) microsatellites are predominant in higher plants (See additional files), in agreement with other SSR survey studies [6,10,11,21] supporting the relative distribution of motifs in these plant groups. However, gymnosperm species showed the lowest SSR occurrence within the derived plant groups. *Pinus *and *Gnetum *results indicate low SSR frequencies as intrinsic characteristics of gymnosperms, such as suggested by other results obtained with distinct methods [10,23,28,29]. The patterns of occurrence of dimers and trimers found in the EST databases of the selected species are shown on Additional files 1 and 2, respectively.

The average GC-content in the 11 datasets was 48.55%. Significantly increased GC-contents were detected for the green algae *Chlamydomonas *(57.22%) and *Mesostigma *(51.36%), for the moss *Syntrichia ruralis *(54.75%) and the fern moss *Sellaginella *spp. (51.38%). These results are in agreement with other genomic comparative analyses of a wide range of plant groups, where the lower groups presented the higher contents [23,31,32]. The remaining species showed similar results (Table 1).

### Dimer and Trimer most frequent motifs

For algae species, the most frequent dimer motifs were AC/GT and CA/TG (Figure 2). For example, in *C. reinhardtii*, from 548 dimer occurrences, 199 AC/GT and 233 CA/TG motifs were found. The predominant trimer motifs found were GCA/TGC, CAG/CTG and GCC/GGC (Additional file 3) with 55, 46 and 39 occurrences in 263 trimers found for algae species. For nonvascular plants, the predominant dimer motifs were AG/CT (239/1,049), AT/AT (226/1,049) and GA/TC (340/1,049), as found for *P. patens*. For mosses, the most frequent trimers found within the studied species were GCA/TGC, AAG/CTT and AGC/GCT. For vascular plants, the most frequent motifs were AG/CT and GA/TC. In *O. sativa*, 246 (43%) and 191(33%) occurrences for these motifs were found, respectively, in a total of 578 dimer occurrences. The GC/GC was only detected in *C. reinhardtii*. There has been a report on the abundance of GC elements in *Chlamydomonas *genome libraries [33].

For the other species this motif has not been reported in high frequencies [10,11,23,28,34].

Among trimer motifs, there was a predominance of AAG/CTT, AGA/TCT, GGA/TCC and GAA/TTC in higher plants. In lower plants, the motifs GCA/TGC and CAG/CTG were predominant. The trimer motif CCG/CGG is predominant in the algae *C. reinhardtii *and the model moss *P. patens*, and could reflect the high GC content in these two species. However, this relationship does not hold for the other cryptogams analysed. The increased CCG/CGG frequency has been described earlier for grasses and has been related to a high GC-content [10]. In this context, the CCG/CGG increase in *Chlamydomonas *and *P. patens *was consistent, but, a previous study reported that it can not be taken as a rule, since higher GC values were found for other lower groups with low CCG/CGG contents [23]. For rice CCG/CGG is the predominant motif and its content appears to be high in the members of the grass family [11,21].

Comparing all plant groups selected for this *in silico *study, the most frequent dimer motifs found were AG/CT and GA/TC, occurring for all plant species. The most frequent trimers were AAG/CTT and GCA/TGC occurring in the 11 studied species.

### Tetramers, Pentamers and Hexamers

Tetramer and pentamer motifs were rare for all studied species except for *M. viride*. This algae showed the higher frequencies in loci formed by motifs longer than three nucleotides with 36.95% of tetramer and 19.56% of pentamer motifs. Although these results are in agreement with other study [23], it is difficult to state that this is a rule for this species, since the EST database size for *Mesostigma *is the smallest one available among the studied databases. In general, tetramer and pentamer motifs predominantly found for *Oryza, Physcomitrella *and *Selaginela *where CATC/GATG, CTCC/GGAG, GATC/GATC, TGCT/AGCA (Additional file 4) and CTTCT/AGAAG, GGAGA/TCTCC, GGCAG/CTGCC, TCTCG/CGAGA and TGCTG/CAGCA (Additional file 5) and these were the most frequent motifs, at least for two out of three of these species.

Hexamer motifs were predominant in novel taxa such as gymnosperms and flowering plants [3,21,35]. *P. taeda *and *G. gnemom *showed the highest frequency (26.95%) of these motifs, but none of the hexamer motifs found in *Gnetum *and *Pinus *were found in common with other plant EST databases. However, one can not state the absence of hexamer motif patterns in plant groups, since in Bryophytes there is a possibility of patterns occurring within closely related groups. For *P. patens *and *M. polymorpha *the AGCAGG/AGCAGG, AGCTGG/CCAGGT, CAGCAA/TTGCTG and TGGTGC/GCACCA motifs occur in both species (Additional file 6). Based on plastid molecular data, Marchantiophyta and Bryophyta originated about 450 Mya [36] and its possible that some repeats are conserved for recently formed groups, but it would be necessary to include others species in further analyses to confirm this hypothesis. For the other SSR types (7, 8, 9 and 10 repeats) frequencies were very low (less than 2 occurrences per motif) and were not further characterized.

### *Physcomitrella patens *SSR loci *versus *Gene Ontology assignments

For the 4,909 SSR loci found for *P. patens *EST sequences, 1,750 had GO assignments. More than 25% of these hits were exclusive to *P. patens*. However, up to 70% of SSR loci were found as conserved across the moss and the higher plant species *O. sativa*, *Vitis vinifera *L. and *A. thaliana*. On Table 3, the distribution of the best Blast hits is presented.

**Table 3:** Distribution of Blast hits for *Physcomitrella patens *SSR loci sequences against several taxa with GO assignment

Taxa	Best Hits (%)
*Physcomitrella patens*	26.90
*Oryza sativa*	10.89
*Vitis vinifera*	10.80
*Arabidopsis thaliana*	9.00
*Populus trichocarpa*	8.60
*Zea mays*	7.18
*Picea sitchensis*	5.60
*Ricinus communis*	4.80
*Glycine max*	3.90
*Sorghum bicolor*	3.90
*Medicago truncatula*	1.48
*Nicotiana tabacum*	0.75
*Solanum tuberosum*	0.63
*Micromonas pusilla*	0.56
*Micromonas sp.*	0.55
*Chlamydomonas reinhardtii*	0.48
*Triticum aestivum*	0.47
*Solanum lycopersicum*	0.46
*Elaeis guineensis*	0.41
*Hordeum vulgare*	0.40
*Ostreococcus lucimarinus*	0.39
*Ostreococcus tauri*	0.35
*Cyanothece sp.*	0.29
*Psium sativum*	0.28
*Brassica rapa*	0.28
*Spinacia oleraceae*	0.25
*Gossypium hirsutum*	0.21
*Pinus contorta*	0.21

**Figure 3:**
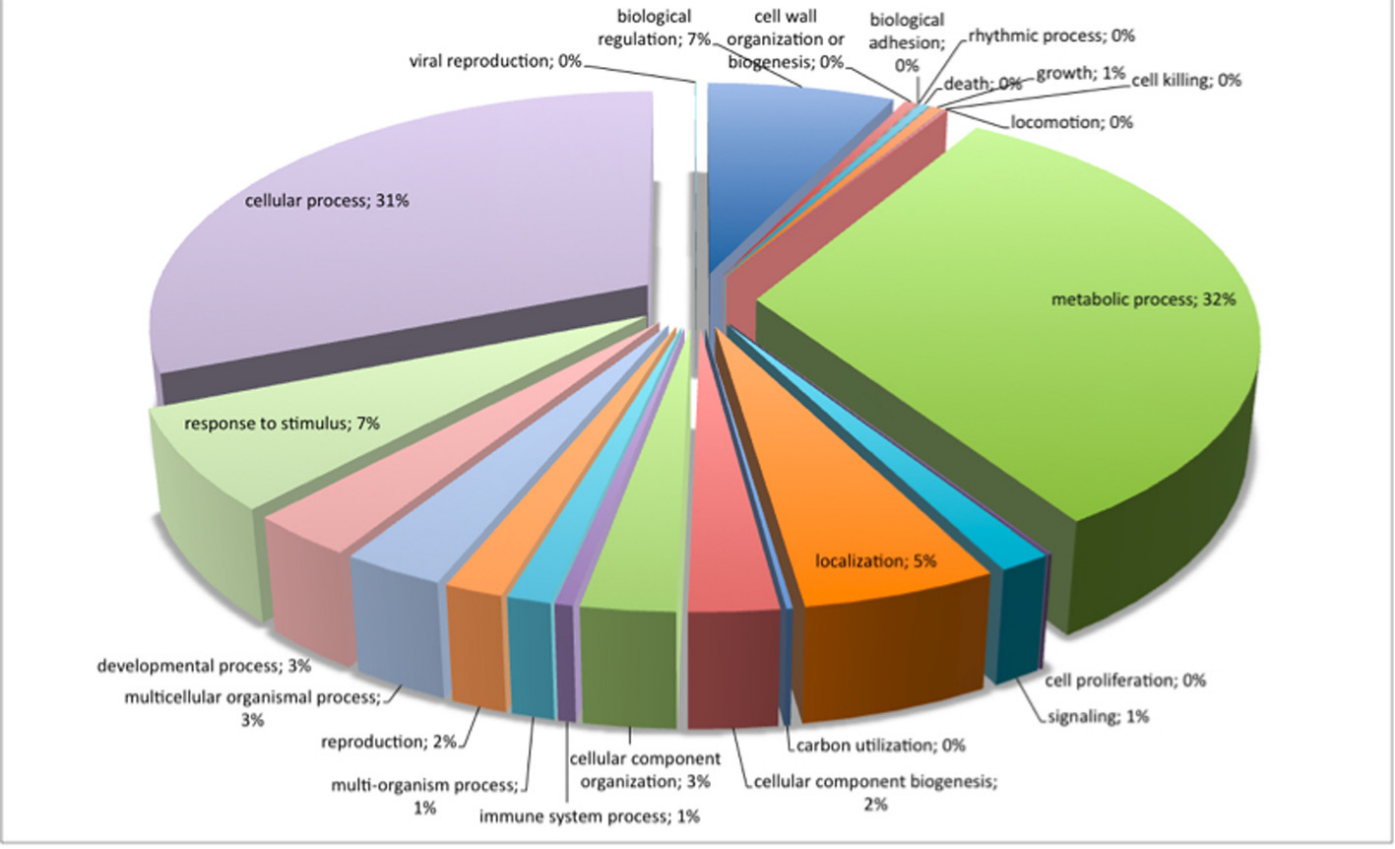
Distribuition of *Physcomitrella patens *SSR loci within sequences of known biological processes in Gene Ontology.

**Figure 4:**
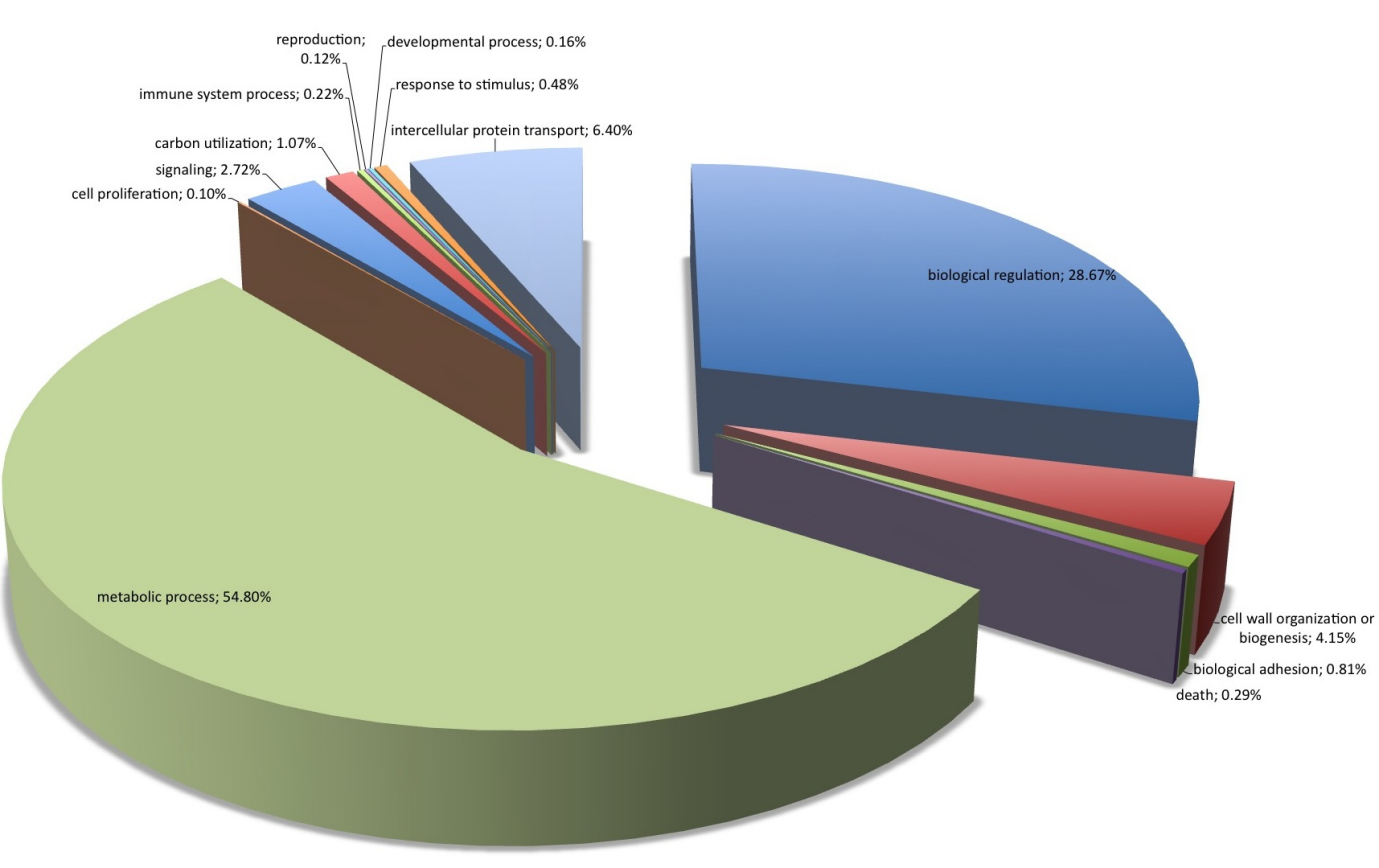
Distribuition of *Physcomitrella patens *genome sequences with Gene Ontology assignments into biological processes. (Data: Rensing et al., 2008).

**Figure 5:**
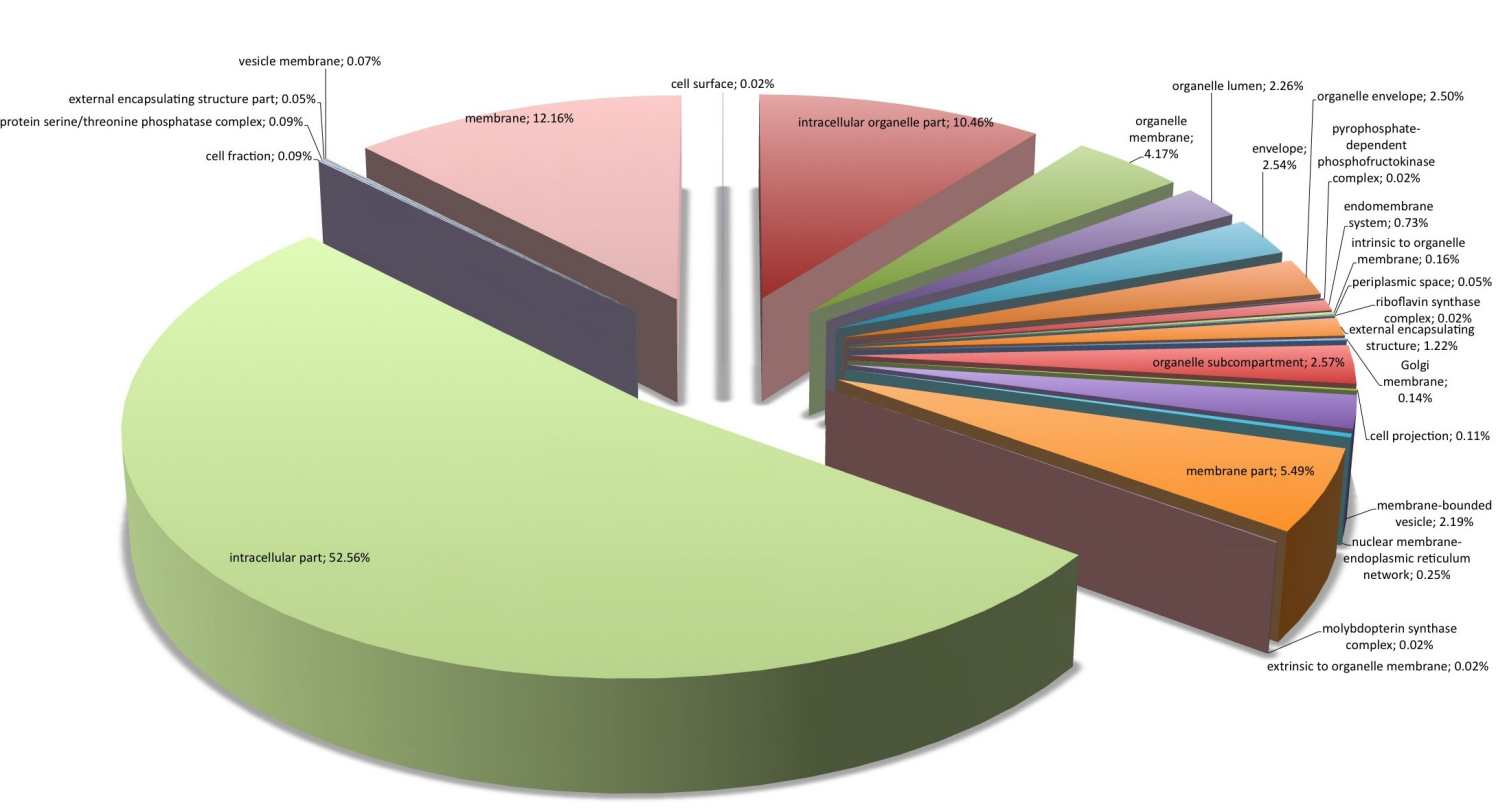
Distribuition of *Physcomitrella patens *SSR loci within sequences of known cellular component in Gene Ontology.

**Figure 6:**
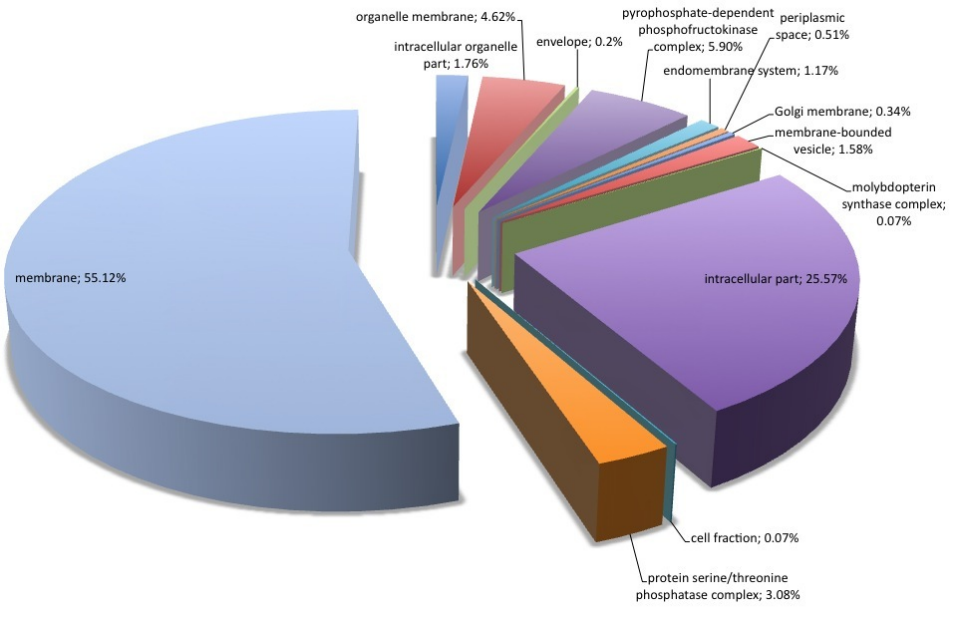
Distribuition of *Physcomitrella patens *genome sequences with Gene Ontology assignments into cellular component. (Data: Rensing et al., 2008).

**Figure 7:**
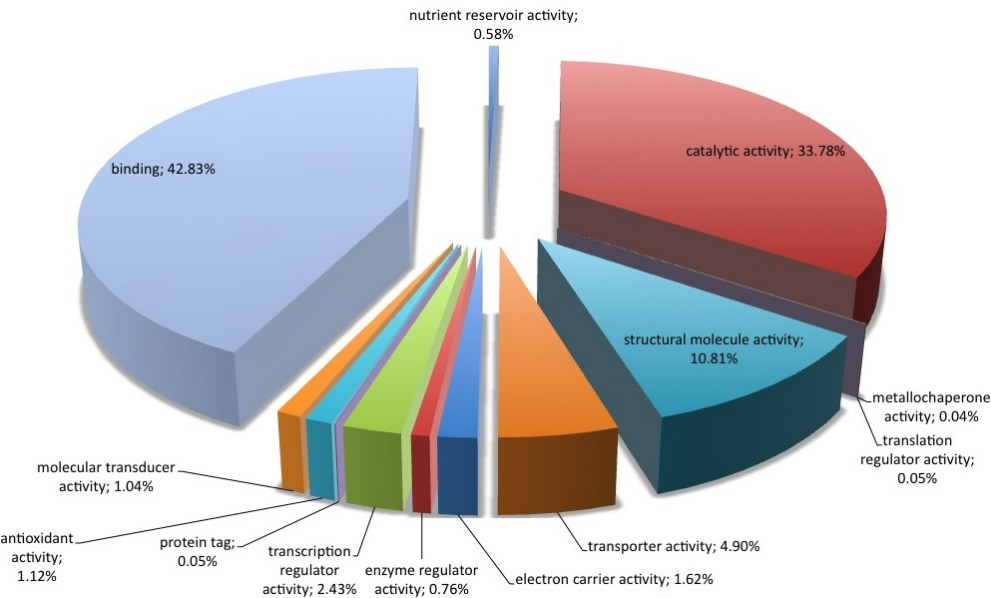
Distribuition of *Physcomitrella patens *SSR loci within sequences of known molecular function in Gene Onthology.

**Figure 8:**
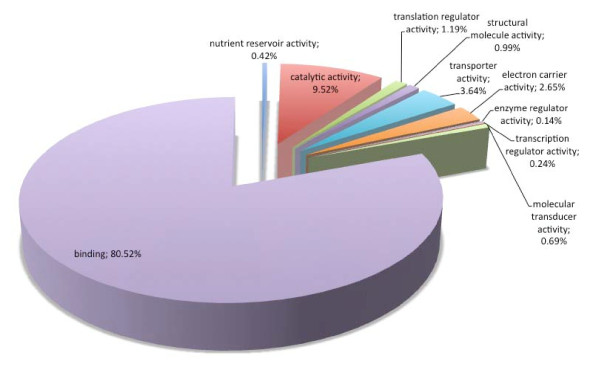
Distribuition of *Physcomitrella patens *genome sequences with Gene Ontology assignments into molecular function. (Data: Rensing et al., 2008).

Regarding biological processes, the majority of SSR loci found were involved with metabolic (32.17%) and cellular (31.02%) processes (Figure 3). Comparing all *P. patens *genome sequences with Gene Ontology assignment and those containing SSRs (Figure 4), there was a concentration of SSRs in metabolic process genes. Biological adhesion, rhythmic processes, growth and cell killing processes had the lowest SSR contents among the *P. patens *transcripts. Similar results were found comparing *P. patens *and *A. thaliana *EST libraries [37]. This author suggested that genes that are involved in protein metabolism and biosynthesis are well conserved between mosses and vascular plants. These patterns were confirmed for mosses using *Syntrichia ruralis *and *P. patens *transcript databases, respectively [38,39]. For cellular components (Figure 5) the majority of SSRs found are related to intracellular component gene sequences (52.52%) and membrane elements (12.15%). This ontology levels were reported as the majority of GO assignments in for *P. patens *annotated sequences [39]. Currently, more than half of cellular component GO annotations for *P. patens *genome [32] are related with membrane structure (Figure 6). Our results show the enrichment of SSR occurrence mainly for genes related to this structural level. The whole genome molecular function assignment level in Gene Ontology revealed a predominance of binding genes (80.51%), suggesting these are representatively higher in *P. patens *genome (Figure 7). However, when EST sequences containing SSRs are assessed with the Gene Ontology assigned molecular function (Figure 8), a relative increase of other functions is revealed. Sequences associated with binding decrease (42.81%), and those related to catalytic activity (33.76%), and structural molecule activity (10.80%) increase. These findings agree to the expectations concerning the cellular function and are consistent with ratios observed for rice, *Arabidopsis*, and for the bryophytes *Syntrichia ruralis *and *P. patens *[32,38-41]. The higher occurrence of SSR loci in this ontology level indicate a good potential for using these molecular markers to saturate pathways associated to those functions described above.

### Predicted coding for SSR loci

**Figure 9:**
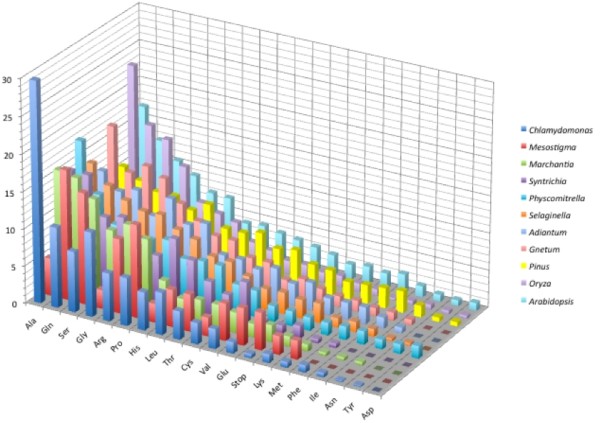
Predicted amino acid occurrences in SSR loci within plant groups studied.

The predicted amino acid content for the SSR loci detected in the eleven species studied is shown in Figure 9. The amino acids arginine (Arg), alanine (Ala) and Serine (Ser) were predominant for all species. Alanine was predominant for the majority of cryptogams, ranging from 14.85% to 29.7%. Exceptions were observed for *Adiantum*, *Mesostigma *and *Physcomitrella*, in which serine (Ser), glutamic acid (Glu) and leucine (Leu) were the predominant amino acid (up to 17%). Serine (up to 11%) was predominant for fern species and for *Gnetum *and *Arabidopsis*, *Pinus *and *Oryza *showed arginine as the predominant amino acid (10.46% and 23.31%, respectively). Tyrosine (Tyr), asparagine (Asp), aspartic acid (Asn) were the amino acids found at lower frequencies among SSR loci for all species and were practically absent in the algae species surveyed. In bryophytes, methionine was only found in *Physcomitrella*, but at a small frequency (1.7%). For all higher plant species databases used in this survey, arginine, alanine, serine, glutamic acid, proline (Pro) and leucine were among the predominant amino acids, agreeing with previous reports for flowering plants [11,3,22,42-45]. No reports were found for amino acid distribution in SSR loci in lower plants.

The small EST databases available for some species did not seem to have hampered the results, since the predicted loci distribution found were consistent within the taxonomic groups. The absence of a relationship between genome size and tandem repeat loci content were reported based in grass genome studies [11], where large genomes such as sugarcane (*Saccharum officinarum *L.), maize and wheat did not present higher frequencies of SSR loci.

#### *Relationship of Codon-bias with EST-SSR motif occurrences*

**Figure 10:**
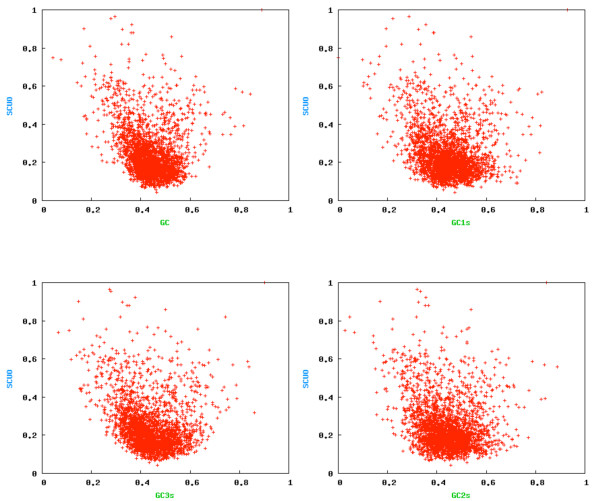
Correlation between synonymous codon usage bias and GC composition in *Physcomitrella patens *EST-SSR sequences.

The high GC-content in some EST-SSR motifs found in the present study can be a result of a codon usage preference by plant species. When we compare the codon usage for the model species included in this study (*Chlamydomonas reinhardtii*, *Physcomitrella patens*, *Oryza sativa *and *Arabidopsis thaliana*) the occurrence of some repeat motifs are reflected in codon-bias known for each species. Higher frequencies of GC were found in the first and third codon position for all four species. However, for the basal plant (*C. reinhardtii*), the preference for GC3 was much higher than the other three species. The first (GC1) and the third (GC3) codon position reached 64.8% and 86.21% of the occurrences, respectively. For rice, GC1 and GC3 frequencies were 58.19% and 61.6%, respectively. For the other model plants, the occurrences at GC3 were lower than the occurrences in GC1, i.e., for *Physcomitrella patens *and *Arabidopsis thaliana*, GC1 (55.49% and 50.84%, respectively) and GC3 (54.6% and 42.4%, respectively) values were found. When one associates these codon usage values with the SSR motif frequencies found, a striking result is obtained for *C. reinhardtii *and rice. In the first, the most frequent motifs were GCA/TGC, CAG/CTG and GCC/GGC and could be explained by the GC1s and GC3s codon preference. In rice the CCG/CGG predominant motif could also be a reflection of GC3s codon preference. For *Arabidopsis*, the most frequent motif found in this study (GAA/TTC) is also the most preferred codon used by this species (GAA) with 34.3% of the occurrences. It also reflects the GC1 preference in the codon usage in this species. In the model moss species the most frequent motifs do not show a relationship with the GC codon usage (Figure 10). Despite the similarities in average codon bias between *P. patens *and *Arabidopsis thaliana*, the distribution pattern is different, with 15% of moss genes being unbiased [46]. An association between the frequency of microsatellite motifs and codon usage could explain the occurrences found in *P. patens*. For example, the most representative motifs GCA/TGC, AAG/CTT and AGC/GCT are also found among the most used codons GCA, AAG and AGC (20.7%, 33.6% and 15%, respectively).

The width of the GC3 distribution in flowering plants was found to be a result of variation in the levels of directional mutation pressure or selection against mutational biases. Likewise, the low frequency of GC2 occurrences is a result of a strong selective pressure against peptide substitution. The balance between these forces could be shaping the distribution of EST-SSR by means of codon usage preference [47].

#### *Positive and negative selection sites in EST-SSR across species*

SSRs represent hyper mutable loci subject to reversible changes in their length [8]. Significant differences in SSR representations exist even among closely related species, suggesting that SSR abundance may change relatively rapidly during evolution [48]. To infer about the selection pressures (dN/dS ratio) on EST-SSR found for the 11 species chosen for this work, we used the common most frequent motif in all species (AAG/CTT and GCA/TGC). The dN-dS test revealed few negatively selected sites in the triplets for each EST-SSR (Additional file 7). The positive selection in SSR based sequence was reported in other studies [8,49-51]. More than 50% of sites for both motifs analyzed across species were under a positive selection (dN/dS > 1), suggesting a weak selection pressure on these EST-SSR motifs, as was reported for other species [52,53]. The occurrence of selective sweeps or background selection in ancestral lineages [54] cannot be discarded, however it could not be tested with the present data.

#### *In silico transferability of EST-SSR across species*

Across-species transferability of EST-SSRs is greater than genomic SSRs, as they originate from expressed regions and therefore they are more conserved across a number of related species [6].

The virtual PCR shows a lower transferability of *Chlamydomonas reinhardtii *EST-SSR for most of the plant species tested. The best results were found for *Adiantum *and *Arabidopsis*, where successful rates of positive EST-SSR amplicons derived from algae were 26% and 9%, respectively. When EST-SSR primers designed from *Arabidopsis *were used against other species, again low transferability rates were found, being the best positive cases found in *Physcomitrella*, *Pinus *and rice with amplification rates of 1.04%, 1.20% and 1.90%. The summary of *in silico *PCR results can be accessed in the Additional files section of this article. Some reports suggest that SSR markers have higher transferability rates when used between closely related species [6,22,55]. In this work virtual PCR amplification did follow the same trend.

**Figure 11:**
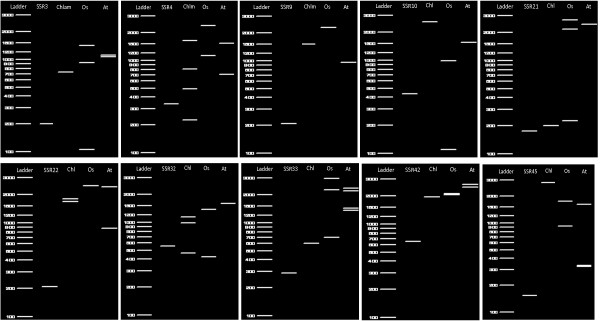
Eletronical eletrophoresis gel for 10 primers set design for *Physcomitrella patens *EST-SSR (SSRn) across *Chlamydomonas reinhardtii *(Chml) *Oryza sativa *(Os) and *Arabidopsis thaliana *(At) EST databases.

For the positive EST-SSRs found for the *in silico *transfer, ten sets of *Physcomitrella *EST-SSR primers were used to illustrate the transferability results using an electronic tool [56] to simulate gel electrophoresis (Figure 11). For the three tested EST-databases only two primers amplified a single locus in each species (SSR9 and SSR10). In the other sets 2, 3 and even 4 virtual amplicons were observed (Additional file 8). For *Chlamydomonas*, 70% of the tested primers resulted in one amplicon and 10% each resulted in 2, 3 or 4 amplifications. However, only 20% of amplicons obtained in this algae species are related to the EST-SSR sequence, suggesting that the majority of designed EST-SSR primers act as degenerate when applied to *Chlamydomonas*. For rice, 30%, 40% and 10% of tested primers resulted in one, two or three amplifications, respectively. In *Arabidopsis *40%, 40% and 20% of tested primers results in one, two or three amplifications, respectively. For both flowering plants, 50% of tested primers amplified moss EST-SSR homologue sequences, showing a high rate of success for transferability across species. These results agree with other studies where the transfer success rates decrease with the increasing evolutionary distance [55,57-60]. The use of this molecular marker across distant taxonomical groups are not impossible, however our findings confirm that only a few retain their EST-SSR homologue sequences, making this effort hardly worthwhile [61].

## Conclusions

These results make it possible to create strategies for transferring molecular markers based on microsatellites from model to orphan species.

Microsatellites were found in all species studied and variable transfer rates were found as a function of genetic distance among taxa. The motifs found are influenced by species codon usage preference. The two most common motifs among the eleven species are under a positive selection pressure. Primers generating one amplicon in the genome of origin may generate multiple amplicons in other taxa and only a few retain their original targeting sequence. The similarities between the results here presented and other initiatives using similar bioinformatics Perl scripts, such as MISA [23], support *SSRLocator *as a useful tool for SSR survey analyses.

## Methods

An exploratory *in silico *analysis of SSRs was made in ESTs databases of 11 taxa, as follows: two unicellular green algae (*Chlamydomonas reinhardtii *Dang, *Mesostigma viride *Lauterborn.), three bryophytes s. l. [*Marchantia polymorpha *L., *Physcomitrella patens *and *Syntricha ruralis *(Hedw.) Weber & Mohr], two ferns (*Selaginella *spp. and *Adiantum capillus-veneris *L.), two gymnosperms (*Gnetum gnemon *L. and *Pinus taeda *L.) and two flowering plants, a monocot (*Oryza sativa*) and a dicot (*Arabidopsis thaliana*). These species were chosen because the amount of available ESTs data in Genbank (NCBI). As these databases may have redundancy, we used the program CAP3 [62] for MacOX, to construct contigs with the sequences and get non-redundant sequences for each database following the default settings.

Taxa data were loaded into the software *SSRLocator *[63], to investigate the presence of tandem repetitive elements (SSRs). The analysis was performed following the search parameters for repetitive elements in class I (≥ 20 bp) described as more efficient molecular markers [17]. Data resulting from *in silico *analyses were assessed for occurrence patterns in chosen taxa databases. The same analysis was performed using MISA script http://pgrc.ipk-gatersleben.de/misa/ software to search for SSR occurrences per contig. Several instructions in the algorithm used in *SSRLocator *resemble those from MISA [19] and SSRIT [17]. However, additional instructions have been inserted in *SSRLocator's *code. Instead of allowing the overlap of a few nucleotides when two SSRs are adjacent to each other and one of them is shorter than the minimum size for a given class as found in MISA and SSRIT, a module written in Delphi language records the data and eliminates such overlaps. For GC content, Perl scripts were used and the results were stored in text files (.txt) for later comparative analyses.

For the predicted amino acid contents in the SSR loci, an additional routine script was written in the *SSRLocator *software. This script determined which amino acids were coded by trimer, hexamer and nonamer motifs found in the EST database analysed [63].

To validate the frequencies obtained using the *SSRLocator *software, the *Physcomitrella patens *EST database was chosen.

This database was run with other SSR search scripts and softwares, such as MISA [19] and SPUTINIK [64], running in SCIROKO package [30], MINE SSR http://www.genome.clemson.edu/resources/online_tools/ssr, SSRIT following the SSR categories defined above [17]. The results were exported into Microsoft Excel spreadsheets (MacOSX-Oficce 2008) and respectively grouped by taxon.

A codon-bias for the model plants included in this research (*Chlamydomonas reinhardtii*, *Physcomitrella patens*, *Oryza sativa *and *Arabidopsis thaliana*) was made comparing with the preferencial codon table for each species available at http://www.kazusa.or.jp/codon/. The sequences containing EST-SSR for *Physcomitrella patens *was submitted to CodonO server [65] to confirm the preferencial codon usage compared with the know codon table for this species. To investigate the selective pressure on the triplets on the EST-SSR which occurs in all studied species a dN-dS statistics [66] was used to verify the synonymous and noun-synonymous substitutions in the preferential codons nearby the repeats chosen using the molecular phylogenetics package MEGA4 [67].

The *Physcomitrella patens *SSR results were run through a Gene Ontology (GO) assignment database in order to assess associations between SSR loci and biological processes, cellular components and molecular function of known genes. A fasta file with all EST-SSRs found in *P. patens *was subjected to Blast2GO software and ran against the GO annotated sequences, and the obtained hits were compiled.

To verify the potential transferability of this molecular markers we have tested *in silico *all EST-SSR found for the plant ancestral lineage, and for the derivative plant group, represented here by the green algae *Chlamydomonas reinhardtii *and *Arabidopsis thaliana*, across the others species EST database used for the present SSR survey. Electronic PCR [68] was used to verify the transferability of EST-SSRs across studied species. The positive results found were used to simulate a gel electrophoresis with aid of SIMGEL.exe included in the SPCR package [56] using the *Physcomitrella patens *EST-SSR sequences to design primers and *Chlamydomonas*, rice and *Arabidopsis *as templates. The virtual amplicons resulted for each primer set tested across species were aligned to verify the homology between the amplicons.

## Authors' contributions

FCV carried out all *in silico *studies, including the SSR survey, the electronic PCR and the sequence alignment for selective sites mining and drafted the manuscript. LCM created the SSR script used and participated in the design of the study. ACO conceived the study, and participated in its design and coordination. All authors read and approved the final manuscript.

## Supplementary Material

Additional file 1 Patterns of occurrence for dimer SSR motifs in percentage. Click here for file

Additional file 2 Patterns of occurrence for trimer SSR motifs in percentage. Click here for file

Additional file 3 Predominant trinucleotide microsatelites motifs loci occurrences per species. Click here for file

Additional file 4 Predominant tetramers microsatelites motifs loci occurrences per species. Click here for file

Additional file 5 Predominant pentamers microsatelites motifs loci occurrences per species. Click here for file

Additional file 6 Predominant hexamers microsatelites motifs loci occurrences per species. Click here for file

Additional file 7 dN/dS table for the common most frequent motifs for 11 species tested EST databases. Click here for file

Additional file 8 Eletronical PCR results table. Click here for file

## References

[B1] Morgante M Olivieri AM PCR-amplified microsatellites as markers in plant genetics The Plant Journal 1993 3 1 175 182 10.1111/j.1365-313X.1993.tb00020.x 8401603

[B2] Jurka J Pethiyagoda C Simple repetitive DNA sequences from Primates: Compilation and analysis Journal of Molecular Evolution 1994 40 120 126 10.1007/BF00167107 7699718

[B3] Tóth G Gáspári Z Jurka J Microsatellites in different eukaryotic genomes: survey and analysis Genome Research 2000 10 967 981 10899146 10.1101/gr.10.7.967 PMC310925

[B4] Iyer RR Pluciennik A Rosche WA Sinder RR Wells RD DNA polymerase III proofreading mutants enhance the expansion and deletion of triplet repeat sequence in Escherichia coli Journal of Biological Chemistry 2000 275 3 2174 2184 10.1074/jbc.275.3.2174 10636923

[B5] Mirkin SM DNA structures, repeat expansions and human hereditary disorders Current Opinion in Structural Biology 2006 16 3 351 358 10.1016/j.sbi.2006.05.004 16713248

[B6] Varshney RK Graner A Sorrells ME Genic microsatellite markers in plants: features and applications Trends in Biotechnology 2005 23 1 48 55 10.1016/j.tibtech.2004.11.005 15629858

[B7] Varshney RK Hoisington DA Tyagy AK Advances in cereal genomics and applications in crop breeding Trends in Biotechnology 2006 24 11 490 499 10.1016/j.tibtech.2006.08.006 16956681

[B8] Kashi Y King DG Simple sequence repeats as advantageous mutators in evolution Trends Genet 2006 22 253 259 10.1016/j.tig.2006.03.005 16567018

[B9] Gupta PK Rustgi S Sharma S Singh R Kumar N Balyan HS Transferable EST-SSR markers for the study of polymorphism and diversity in bread wheat Molecular Genetics and Genomics 2003 270 315 323 10.1007/s00438-003-0921-4 14508680

[B10] Morgante M Hanafey M Powell W Microsatellites are preferentially associated with nonrepetitive DNA in plant genomes Nature Genetics 2002 3 2 194 200 10.1038/ng822 11799393

[B11] Maia LC Souza VQ Kopp MM Carvalho FIF Oliveira AC Tandem repeat distribution of gene transcripts in three plant families Genetics and Molecular Biology 2009 32 4 1 12 10.1590/S1415-47572009005000091 PMC3036893 21637460

[B12] Subramanian S Mishra RK Singh L Genome-wide analysis of microsatellite repeats in humans: their abundance and density in specific genomic regions Genome Biology 2003 4 2 R13 10.1186/gb-2003-4-2-r13 12620123 PMC151303

[B13] Li YC Korol AB Fahima T Beiles A Nevo E Microsatellites: genomic distribution, putative functions and mutational mechanisms: a review Molecular Ecology 2002 11 2453 2465 10.1046/j.1365-294X.2002.01643.x 12453231

[B14] Marcotte EM Pellegrini M Yeates TO Eisenberg D A census of protein repeats Journal of Molecular Biology 1999 293 151 10.1006/jmbi.1999.3136 10512723

[B15] Kashi Y King D Soller M Simple sequence repeats as a source of quantitative genetic variation Trends in genetics 1997 13 74 78 10.1016/S0168-9525(97)01008-1 9055609

[B16] Wren JD Forgacs E Fondon JW III Pertsemlidis A Cheng SY Gallardo T Williams RS Shohet RV Minna JD Garner HR Repeat polymorphisms within gene regions: phenotypic and evolutionary implications American Journal of Human Genetics 2000 67 345 356 10.1086/303013 10889045 PMC1287183

[B17] Temnykh S DeClerck G Lukashova A Lipovich L Cartinhour S McCouch S Computational and experimental analysis of microsatellites in rice (*Oryza sativa *L.): frequency, length variation, transposon associations, and genetic marker potential Genome Research 2001 11 8 1441 52 10.1101/gr.184001 11483586 PMC311097

[B18] McCouch SR Teytelman L Xu Y Development and mapping of 2240 new SSR markers for rice (*Oryza sativa *L.) DNA research 2002 9 6 199 207 10.1093/dnares/9.6.199 12597276

[B19] Thiel T Michalek W Varshney RK Graner A Exploiting EST databases for the development of cDNA derived microsatellite markers in barley (*Hordeum vulgare *L.) Theoretical and Applied Genetics 2003 1-6 411 422 10.1007/s00122-002-1031-0 12589540

[B20] Nicot N Chiquet V Gandon B Amilhat L Legeai F Leroy P Bernard M Sourdille P Study of simple sequence repeat (SSR) markers from wheat expressed sequence tags (ESTs) Theoretical and Applied Genetics 2004 1-9 4 8008 5 10.1007/s00122-004-1685-x 15146317

[B21] Lawson MJ Zhang L Distinct patterns of SSR distribution in the *Arabidopsis thaliana *and rice genomes Genome Biology 2006 7 R14 3 10.1186/gb-2006-7-2-r14 16507170 PMC1431726

[B22] Zhang L Yuan D Yu S Li Z Cao Y Miao Z Qian H Tang K Preference of simple sequence repeats in coding and non coding regions of *Arabidopsis thaliana* Bioinformatics 2004 20 1081 1086 10.1093/bioinformatics/bth043 14764542

[B23] von Stackelberg MV Rensing SA Reski R Identification of genic moss SSR markers and a comparative analysis of twnty-four algal and plant gene indices reveal species-specific rather than group-specific characteristics of microsatellites BMC Plant Biology 2006 6 9 10.1186/1471-2229-6-9 16734891 PMC1526434

[B24] Cordeiro GM Casu R McIntyre CL Manners JM Henry RJ Microsatellite markers from sugarcane (Saccharum spp.) ESTs cross transferable to erianthus and sorghum Plant science 2001 16 6 1115 1123 10.1016/S0168-9452(01)00365-X 11337068

[B25] Kantety RV La Rota M Matthews DE Sorrells ME Data mining for simple sequence repeats in expressed sequence tags from barley, maize, rice, sorghum and wheat Plant molecular biology 2002 48 5-6 5-1 11 10.1023/a:1014875206165 11999831

[B26] Asp T Frei UK Didion T Nielsen KK Lübberstedt T Frequency, type, and distribution of EST-SSRs from three genotypes of *Lolium perenne*, and their conservation across orthologous sequences of *Festuca arundinacea*, *Brachypodium distachyon*, and *Oryza sativa* BMC plant biology 2007 12 7 36 10.1186/1471-2229-7-36 PMC1950305 17626623

[B27] Echt CS May-Marquardt P Hseih M Zahorchak R Characterization of microsatellire markers in eastern white pine Genome 1996 39 1102 1108 10.1139/g96-138 8983182

[B28] Echt CS May-Marquardt P Survey of microsatellite DNA in pine Genome 1997 40 9 17 10.1139/g97-002 9061909

[B29] Fisher PJ Gardner RC Richardson TE Single locus microsatellites isolated using 5'anchored PCR Nucleic Acids Research 1996 24 4369 4372 10.1093/nar/24.21.4369 8932400 PMC146250

[B30] Kofler R Schlotterer C Lelley T SciRoKo: A new tool for whole genome microsatellite search and investigation Bioinformatics 2007 23 1683 1685 10.1093/bioinformatics/btm157 17463017

[B31] Qiu Y-L Lee J Bernasconi-Quadroni B Soltis DE The earliest Angiosperms: Evidence from mitochondrial, palstid and nuclear genomes Nature 1999 402 404 407 10.1038/46536 10586879

[B32] Rensing SA Lang D Zimmer AD The *Physcomitrella *genome reveals insights into the conquest of land by plants Science 2008 319 64 69 10.1126/science.1150646 18079367

[B33] Wakarchuk WW Müller FW Beck C F. Two GC-rich elements of *Chlamydomonas reinhardtii *with complex arrangements of directly repeated sequences motifs Plant Molecular Biology 1992 18 143 146 10.1007/BF00018468 1731966

[B34] Yashoda R Sumathi R Chezhian P Kavitha S Ghosh M *Eucalyptus *microsatellites mined *in silico*: survey and evaluation Journal of Genetics 2008 87 1 21 25 10.1007/s12041-008-0003-9 18560170

[B35] Jiang D Zhong GY Hong QB Analysis of microsatellites in citrus unigenes Acta genetica Sinica 2006 33 4 345 53 10.1016/S0379-4172(06)60060-7 16625833

[B36] Magallón S Hilu KW S. B. Hedges, S. Kumar Land plants (Embryophyta) The Timetree of Life 2009 Oxford, University Press 133 137

[B37] Nishiyama T Fujita T Shin-I T Seki M Nishide H Uchiyama I Kamiya A Carninci P Hayashizaki Y Shinozaki K Kohara Y Hasebe M Comparative genomics of Physcomitrella patens gametophytic transcriptome and Arabidopsis thaliana: Implication for land plant evolution PNAS 2003 100 13 8007 8012 10.1073/pnas.0932694100 12808149 PMC164703

[B38] Oliver MJ Dowd SE Zaragoza J Mauget SA Payton PR The rehydration transcriptome of the desiccation-tolerant bryophyte Tortula ruralis: Transcript classification and analysis BMC Genomics 2004 5 89 10.1186/1471-2164-5-89 15546486 PMC535811

[B39] Lang D Eisinger J Reski R Resing SA Representation and High-Quality Annotation of the Physcomitrella patens Transcriptome Demonstrates a High Proportion of Proteins Involved in Metabolism in Mosses Plant Biology 2005 7 238 250 10.1055/s-2005-837578 15912443

[B40] Ware D Jaiswal P Ni J Pan X Chang K Clark K Teytelman L Schmidt S Zhao W Cartinhour S McCouch S Stein L Gramene: a resource for comparative grass genomics Nucleic Acids Research 2002 30 103 105 10.1093/nar/30.1.103 11752266 PMC99157

[B41] Rhee SY Beavis W Berardini TZ The Arabidopsis Information Resource (TAIR): a model organism database providing a centralized, curated gateway to Arabidopsis biology, research materials and community Nucleic Acids Research 2003 31 224 228 10.1093/nar/gkg076 12519987 PMC165523

[B42] Jung S Abbott A Jesudurai C Tomkins J Main D Frequency, type, distribution and annotation of simple sequence repeats in Rosaceae ESTs Functional & integrative genomics 2005 5 3 136 43 10.1007/s10142-005-0139-0 15761705

[B43] La Rota M Kantety RV Yu JK Sorrells ME Nonrandom distribution and frequencies of genomic and EST-derived microsatellite markers in rice, wheat, and barley BMC Genomics 2007 18 1 23 6 10.1186/1471-2164-6-23 PMC550658 15720707

[B44] Varshney RK Thiel T Stein N Langridge P Graner A In silico analysis on frequency and distribution of microsatellites in ESTs of some cereal species Cell Mol Biol Lett 2002 7 537 546 12378259

[B45] Parida SK Anand Raj Kumar K Dalal V Singh NK Mohapatra T Unigene derived microsatellite markers for the cereal genomes Theor Appl Genet 2006 112 808 817 10.1007/s00122-005-0182-1 16429310

[B46] Rensing SA Fritzomsky D Lang D Reski R Protein encoding genes in an ancient plant: analysis of codon usage, retained genes and splice sites in a moss, *Physcomitrella patens* BMC genomics 2005 6 43 10.1186/1471-2164-6-43 15784153 PMC1079823

[B47] Kawabe A Miyashita NT Patterns of codon usage bias in three dicot an four monocot plant species Genes and Genetic System 2003 78 343 352 10.1266/ggs.78.343 14676425

[B48] Mrázek J Analysis of distribuition indicates diverse functions of simple sequence repeats in *Mycoplasma *genomes Molecular Biology and Evolution 2006 23 1370 1385 16618962 10.1093/molbev/msk023

[B49] King DG Kashi Y Indirect selection for mutuability Heredity 2007 99 123 124 10.1038/sj.hdy.6800998 17507902

[B50] King DG Soller M Wasser SP Variation and fidelity: The evolution of simple sequence repeats as functional elements in adjustable genes Evolutionary Theory and Processes: Modern Perspectives 1999 Kluwer Academic Publisher, the Netherlands 65 82

[B51] Vigouroux Y Matsuoka Y Doebley J Directional evolution for microstellites size in maize Molecular Biology and Evolution 2003 20 1480 1483 10.1093/molbev/msg156 12832640

[B52] Ellis JR Burke JM EST-SSRs as a resource for population genetic analyses Heredity 2007 99 125 132 10.1038/sj.hdy.6801001 17519965

[B53] Yatabe Y Kane NC Scotti-Saintagne C Rieseberg LH Rampant gene exchange across a strong reproductive barrier between the annual sunflowers, *Helianthus annuus *and *H petiolaris* Genetics 2007 175 1883 1893 10.1534/genetics.106.064469 17277373 PMC1855124

[B54] Wrigth SI Gaut BS Molecular population genetics and the search for adaptative evolution in plants Molecular Biology and Evolution 2005 22 3 506 519 15525701 10.1093/molbev/msi035

[B55] Chapman MA Hvala J Strever J Development, polymorphism, and cross-taxon utility of EST-SSR markers from safflower (*Carthamus tinctorius *L.) Theoretical and Applied Genetics 2009 120 85 91 10.1007/s00122-009-1161-8 19820913

[B56] Cao Y Wang L XU K Kou C Zhang Y Wei G He J Wang Y Zhao L Information theory-based algorithm for *in silico *prediction of PCR products with whoke genomic sequences as templates BMC bioinformatics 2005 6 190 10.1186/1471-2105-6-190 16042814 PMC1183192

[B57] Brondani C Rangel PHN Borba TCO Brondani RPV Transferability of microsatellite and sequence tagged site markers in *Oryza *species Hereditas 2003 138 187 192 10.1034/j.1601-5223.2003.01656.x 14641482

[B58] Castillo A Budak H Varshney RK Dorado G Graner A Hernandez P Tranferability and polimorphism of barley EST-SSR markersused for phylogenetic analysus in *Hordeum chilense* BMC plant biology 2008 8 97 10.1186/1471-2229-8-97 18822176 PMC2569940

[B59] Yodav OP Mitchell SE Fulton TM Kresovich S Tranferring molecular markers from sorghum, rice and other cereals to pearl millet and identifying polumorphic markers Journal of SAT Agricultural Research 2008 6 1 4

[B60] Zeid M Yu JK Goldowitz I Denton ME Cross-amplification of EST-derived markers among 16 grass species Field Crops Research 2010 118 28 35 10.1016/j.fcr.2010.03.014

[B61] Barbará T Palma-Silva C Paggi GM Bered F Fay MF Lexer C Cross-species transfer of nuclear microsatellites markers: potential and limitations Molecular Ecology 2007 16 3759 3767 17850543 10.1111/j.1365-294X.2007.03439.x

[B62] Huang X Madan A CAP3: A DNA sequence assembly program Genome Research 1999 9 868 877 10.1101/gr.9.9.868 10508846 PMC310812

[B63] Maia LC Palmieri DA Souza VQ Kopp MM Carvalho FIF Oliveira AC SSR Locator: Tool for Simple Sequence Repeat Discovery Integrated with Primer Design and PCR Simulation International Journal of Plant Genomics 2008 Article ID 412696, 9 pages 18670612 10.1155/2008/412696 PMC2486402

[B64] Abajan C SPUTINIK 1994 http://espressosoftware.com/sputnik/index.html

[B65] Angellotti MC Bhuiyan SB Chen G Wan X-F CodonO: codon usage bias analysis within and across genomes Nucleic Acids Research 2007 35 W132 W136 10.1093/nar/gkm392 17537810 PMC1933134

[B66] Yang Z Bielawski JP Statistical methods for detecting molecular adaptation Trends in Ecology and Evolution 2000 12 496 503 10.1016/S0169-5347(00)01994-7 PMC7134603 11114436

[B67] Tamura K Dudley J Nei M Kumar S MEGA4: Molecular Evolutionary Genetics Analysis (MEGA) software version 4.0 Molecular Biology and Evolution 2007 24 1596 1599 10.1093/molbev/msm092 17488738

[B68] Schuler GD Sequence mapping by eletronic PCR Genome Research 1997 7 5 541 550 9149949 10.1101/gr.7.5.541 PMC310656

