# Supplementary material for: In silico comparative analysis of SSR markers in plants
Source: BMC Plant Biol. 2011 Jan 19;11:15. doi: 10.1186/1471-2229-11-15 (PMC3037304; doi:10.1186/1471-2229-11-15)
Supplement: Additional file 1 — Patterns of occurrence for dimer SSR motifs in percentage. [file 1471-2229-11-15-S1.DOC]

**Additional file 1.** Patterns of occurrence for dimer SSR motifs in percentage.

| *Repeat Motifs* | *Algae* | | *Bryophya l. s.* | | | *Vascular plants* | | | | | |
| --- | --- | --- | --- | --- | --- | --- | --- | --- | --- | --- | --- |
|  |  |  |  |  |  | *Ferns* | | *Gymnosperms* | | *Flowering plants* | |
| *Dimers* | *C. reinhardtii* | *M. viride* | *M. polymorpha* | *S. ruralis* | *P. patens* | *Selaginella spp.* | *A. capillus-veneris* | *G. gnemon* | *P. taeda* | *O. sativa* | *A. thaliana* |
| AC/GT | 36.2 | - | 24.0 | - | 3.8 | 8.1 | 9.0 | - | - | 4.0 | 2.4 |
| AG/CT | 4.4 | 29.3 | 36.0 | 30.4 | 22.8 | 40.3 | 39.5 | 21.4 | 13.4 | 42.6 | 38.3 |
| AT/AT | 2.2 | - | - | - | 21.5 | 2.7 | 0.1 | 14.3 | 41.0 | 9.2 | 8.9 |
| CA/TG | 42.4 | - | 12.0 | - | 5.4 | 6.0 | 15.0 | - | 0.9 | 3.8 | 1.9 |
| GA/TC | 8.5 | 67.2 | 28.0 | 69.6 | 32.4 | 41.6 | 36.1 | 35.7 | 11.5 | 33.1 | 38.5 |
| TA/TA | 5.5 | - | - | - | 14.0 | 1.3 | 0.3 | 28.6 | 32.7 | 7.3 | 10.0 |
| GC/GC | 0.5 | - | - | - | - | - | - | - | - | - | - |
| CT/AG | 0.4 | 3.4 | - | - | - | - | - | - | - | - | - |
