# Supplementary material for: In silico comparative analysis of SSR markers in plants
Source: BMC Plant Biol. 2011 Jan 19;11:15. doi: 10.1186/1471-2229-11-15 (PMC3037304; doi:10.1186/1471-2229-11-15)
Supplement: Additional file 2 — Patterns of occurrence for trimer SSR motifs in percentage. [file 1471-2229-11-15-S2.DOC]

**Additional file 2.** Patterns of occurrence for trimer SSR motifs in percentage.

| *Repeat Motifs* | *Algae* | | *Bryophyta l. s.* | | | *Vascular plants* | | | | | |
| --- | --- | --- | --- | --- | --- | --- | --- | --- | --- | --- | --- |
|  |  |  |  |  |  | *Ferns* | | *Gymnosperms* | | *Flowering plants* | |
| *Dimers* | *C. reinhardtii* | *M. viride* | *M. polymorpha* | *S. ruralis* | *P. patens* | *Selaginella spp.* | *A. capillus-veneris* | *G. gnemon* | *P. taeda* | *O. sativa* | *A. thaliana* |
| AAC/GTT | - | - | - | - | 4.4 | 0.8 | 10.3 | - | 3.3 | 0.3 | 3.9 |
| AAG/CTT | 0.5 | 16.7 | 3,2 | 14.5 | 11.1 | 3.8 | 12.8 | 10,9 | 6.7 | 2.6 | 15.9 |
| AAT/ATT | - | - | - | - | 1.4 | 1.5 | - | - | 10.0 | 0.5 | 0.3 |
| ACA/TGT | 1.6 | - | 3,2 | 1.6 | 5.2 | 1.7 | - | - | - | 0.2 | 4.5 |
| ACC/GGT | - | - | - | - | 0.5 | 2.1 | - | - | - | 2.3 | 1.3 |
| ACG/CGT | - | - | - | - | 1.1 | 1.5 | - | - | - | 1.4 | - |
| ACT/AGT | - | 16.7 | - | - | 1.6 | 0.4 | - | - | 1.1 | 0.2 | 1.3 |
| AGA/TCT | 0.5 | - | - | 4.8 | 8.3 | 1.9 | 17.9 | 7,9 | 5.6 | 2.6 | 22.9 |
| AGC/GCT | - | - | 22,6 | 37.1 | 1.9 | 14.1 | 10.3 | 15,8 | 3.3 | 2.6 | 0.5 |
| AGG/CCT | - | - | - | 12.9 | 1.9 | 1.5 | - | 3,0 | 5.6 | 4.6 | 1.1 |
| ATA/TAT | - | - | - | - | 0.9 | 0.2 | - | 1,0 | 7.8 | 0.3 | - |
| ATC/GAT | - | - | 1,6 | - | 0.9 | - | - | 1,0 | - | 0.6 | 6.3 |
| ATG/CAT | 2.2 | - | - | - | 1.1 | - | 2.6 | - | - | 0.5 | 6.7 |
| CAA/TTG | 0.5 | 16.7 | 6,5 | 8.1 | 1.1 | - | 2.6 | 4,0 | 3.3 | 0.5 | 4.2 |
| CAC/GTG | 4.3 | - | 8,1 | - | 6.9 | 0.8 | 2.6 | 2,0 | 2.2 | 1.8 | 0.6 |
| CAG/CTG | 25.0 | - | 8,1 | 30.6 | 2.7 | 3.2 | - | 16,8 | 13.3 | 3.3 | 0.4 |
| CAT/ATG | - | 16.7 | 1,6 | 1.6 | 5.5 | 20.0 | 7.7 | 6,9 | 2.2 | - | - |
| CCA/TGG | 3.3 | - | - | - | - | - | - | - | 3.3 | 2.5 | 0.8 |
| CCG/CGG | - | - | - | - | 1.7 | 6.8 | - | 1,0 | - | 20.9 | 0.2 |
| CGA/TCG | - | - | 6,5 | 9.7 | 0.2 | 4.6 | - | - | - | 2.9 | - |
| CGC/GCG | 8.7 | - | - | - | 2.0 | 2.5 | - | - | 1.1 | 18.7 | - |
| CTA/TAG | - | - | 1,6 | 1.6 | 0.2 | - | - | - | - | 0.2 | - |
| CTC/GAG | - | - | - | 30.6 | - | - | - | 4,0 | - | 6.8 | 1.0 |
| GAA/TTC | - | 16.7 | 3,2 | 12.9 | 0.8 | 0.2 | - | 5,0 | 7.8 | 2.6 | 17.8 |
| GAC/GTC | - | - | - | 3.2 | 5.3 | 2.7 | 12.8 | - | 4.4 | 1.9 | - |
| GCA/TGC | 29.3 | 16.7 | 24,2 | 40.3 | 8.9 | 4.9 | 15.4 | 15,8 | 7.8 | 2.6 | 0.3 |
| GCC/GGC | 21.2 | - | - | - | 1.3 | 0.8 | 2.6 | - | - | 11.1 | - |
| GGA/TCC | 1.1 | - | - | 11.3 | 8.6 | 15.6 | - | 4,0 | 6.7 | 4.7 | 2.6 |
| GGC/GCC | - | - | 4,8 | - | 1.7 | 4.9 | - | - | - | - | 0.1 |
| GTA/TAC | - | - | 1,6 | 3.2 | 8.0 | 2.9 | - | - | - | 0.1 | - |
| GTT/ACC | - | - | 1,6 | 1.6 | 0.8 | 0.2 | 2.6 | - | - | - | - |
| TAA/TTA | - | - | - | - | - | - | - | - | 3.3 | 0.4 | 1.0 |
| TCA/TGA | - | - | - | 1.6 | 4.4 | 0.6 | - | 1,0 | - | 0.6 | 6.2 |
| TGA/TCA | 1.6 | - | 1,6 | - | - | - | - | - | 1.1 | - | - |
