# Supplementary material for: In silico comparative analysis of SSR markers in plants
Source: BMC Plant Biol. 2011 Jan 19;11:15. doi: 10.1186/1471-2229-11-15 (PMC3037304; doi:10.1186/1471-2229-11-15)
Supplement: Additional file 6 — Predominant hexamers microsatelites motifs loci occurrences per species. [file 1471-2229-11-15-S6.DOC]

**
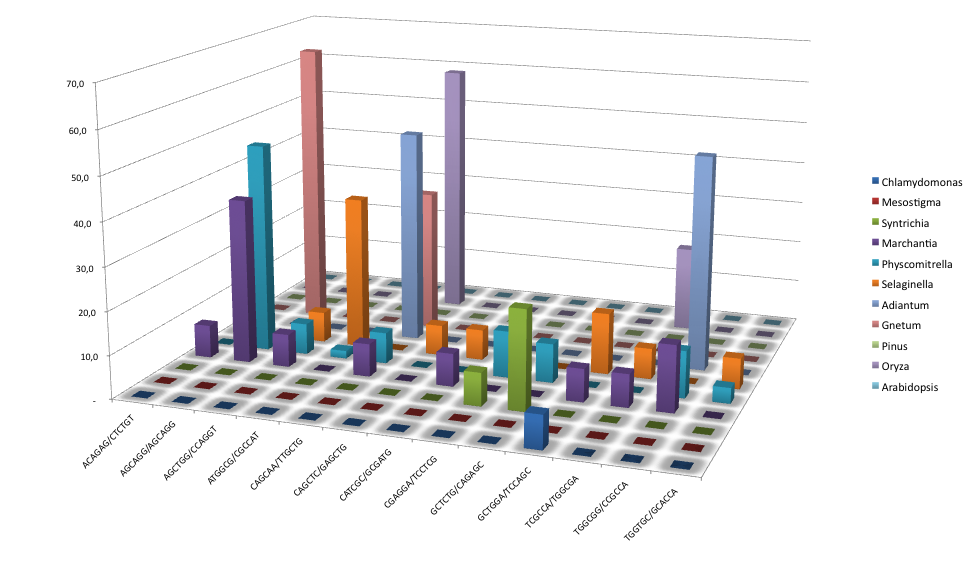
**

SSR (%)

**Additional file 6.** Predominant loci containing hexamers microsatellites motifs per species.
