# Supplementary material for: In silico comparative analysis of SSR markers in plants
Source: BMC Plant Biol. 2011 Jan 19;11:15. doi: 10.1186/1471-2229-11-15 (PMC3037304; doi:10.1186/1471-2229-11-15)
Supplement: Additional file 7 — dN/dS table for the common most frequent motifs for 11 species tested EST databases. [file 1471-2229-11-15-S7.DOC]

**Additional file 7.** dN/dS table for the common most frequent motifs for 11 species tested EST databases.

| **AGG/CCT motif** | | | | | |
| --- | --- | --- | --- | --- | --- |
| Triplet | Syn sites (S) | Nonsyn sites (N) | dN-dS | Prob. (positive selection) | Normalized dN-dS |
| AAG | 0.50454 | 2.23825 | -1.73012 | 0.878433 | -0.187245 |
| CGG | 0.557038 | 2.20646 | 1.08103 | 0.532335 | 0.116996 |
| GAG | 0.501536 | 2.23775 | -0.206366 | 0.771498 | -0.0223344 |
| AGA | 0.513401 | 2.23966 | 0.73118 | 0.614198 | 0.0791335 |
| AAG | 0.519388 | 2.22273 | 0.899794 | 0.657054 | 0.097382 |
| AAG | 0.514282 | 2.19635 | -4.46748 | 0.985997 | -0.483502 |
| AAG | 0.516528 | 2.18331 | 2.74813 | 0.279682 | 0.297422 |
| AAG | 0.731956 | 2.1026 | -0.293794 | 0.690423 | -0.0317965 |
| AAG | 0.547747 | 2.21259 | 1.69983 | 0.462505 | 0.183967 |
| AAG | 0.605001 | 2.23091 | 0.420291 | 0.597842 | 0.0454869 |
| AAG | 0.925331 | 2.05725 | -0.578783 | 0.683643 | -0.06264 |
| AAG | 0.578969 | 2.2423 | 1.53503 | 0.470588 | 0.166131 |
| AGG | 0.518176 | 2.32726 | 3.49594 | 0.276573 | 0.378355 |
| AGA | 0.918584 | 2.07523 | 0.160841 | 0.596693 | 0.0174073 |
| GAG | 0.688006 | 2.19088 | 2.07179 | 0.384344 | 0.224223 |
| CAT | 0.486148 | 2.45053 | 3.24799 | 0.299807 | 0.351521 |
| GAA | 0.865831 | 2.08804 | -0.290612 | 0.63424 | -0.0314521 |
| ATA | 0.910296 | 2.04134 | -1.99686 | 0.815704 | -0.216115 |
| GAT | 0.86488 | 2.09436 | -1.2077 | 0.746108 | -0.130706 |
| GCA | 0.799125 | 1.99794 | 1.50121 | 0.439544 | 0.162472 |
| GCG | 0.542355 | 2.2672 | 0.462816 | 0.5982 | 0.0500892 |
| GCA | 0.957027 | 1.96204 | -1.68236 | 0.809953 | -0.182077 |
| GGC | 0.720549 | 2.11616 | -1.75598 | 0.805406 | -0.190045 |
| GAT | 0.533094 | 2.41637 | 2.67645 | 0.333447 | 0.289664 |
| GGA | 0.590975 | 2.22215 | 4.62304 | 0.182718 | 0.500339 |
| GAT | 0.675461 | 2.08465 | -0.676259 | 0.686507 | -0.0731896 |
| GAA | 0.977162 | 1.96976 | -2.07882 | 0.853884 | -0.224985 |
| GCA | 0.621939 | 2.14384 | 1.60246 | 0.46101 | 0.17343 |
| GCT | 0.590266 | 2.13786 | 3.45118 | 0.231302 | 0.373511 |
| GGT | 0.739893 | 2.14588 | -0.443171 | 0.685264 | -0.0479631 |
| GGT | 0.607556 | 2.28413 | -0.454509 | 0.677626 | -0.0491902 |
| AAT | 0.695619 | 2.26213 | 0.438593 | 0.577958 | 0.0474677 |
| GCA | 0.958111 | 2.01635 | 2.05035 | 0.338642 | 0.221903 |
| GCG | 0.895376 | 2.07833 | -0.97875 | 0.712776 | -0.105927 |
| TGC | 0.623205 | 2.16294 | 3.95243 | 0.213227 | 0.42776 |
| CCG | 0.573093 | 2.37609 | -0.666765 | 0.696335 | -0.072162 |
| CTC | 0.962781 | 2.01998 | -1.50465 | 0.79495 | -0.162844 |
| ATG | 0.81405 | 2.01054 | 1.47434 | 0.441878 | 0.159563 |
| ATG | 0.93283 | 2.03281 | -1.71463 | 0.813332 | -0.185569 |
| CTG | 0.863353 | 2.09092 | 2.88078 | 0.270948 | 0.311778 |
| CCT | 1.0461 | 1.88006 | 0.647174 | 0.522228 | 0.0700417 |
| CTT | 0.837426 | 1.8965 | -2.08193 | 0.810116 | -0.225321 |
| TTC | 0.999669 | 1.78106 | -7.41542 | 0.995243 | -0.80255 |
| CAG | 0.650949 | 2.18821 | 2.24626 | 0.381008 | 0.243106 |
| TCC | 0.839913 | 1.93401 | 0.588486 | 0.54537 | 0.0636901 |
| TTG | 0.654223 | 2.30919 | 0.602481 | 0.561919 | 0.0652048 |
| CCG | 0.671886 | 2.25946 | -2.77437 | 0.859282 | -0.300262 |
| TCG | 0.690428 | 2.2716 | 3.77037 | 0.194335 | 0.408057 |
| CGC | 0.504898 | 2.45568 | -0.647944 | 0.714364 | -0.070125 |
| GCC | 0.805767 | 2.09712 | 0.374755 | 0.567291 | 0.0405587 |
| GGC | 0.484084 | 2.05504 | 3.56397 | 0.326727 | 0.385718 |
| TTA | 0.625928 | 2.1657 | 0.0739089 | 0.621917 | 0.00799894 |
| TAC | 1.18427 | 1.75413 | 1.61597 | 0.37398 | 0.174892 |
| ACT | 0.795257 | 2.02584 | 2.2628 | 0.351983 | 0.244896 |
| CTC | 0.597505 | 2.10293 | 1.39564 | 0.486205 | 0.151046 |
| ACA | 0.99943 | 1.97614 | -1.71936 | 0.851214 | -0.186081 |
| CTT | 0.750545 | 2.18723 | 1.04189 | 0.498332 | 0.112761 |
| CCT | 0.738113 | 1.95809 | -0.979003 | 0.705654 | -0.105955 |
| CCC | 0.831824 | 1.86786 | -1.85934 | 0.805013 | -0.201231 |
| CAT | 0.748215 | 2.0141 | 0.878961 | 0.523515 | 0.0951273 |
| TTC | 0.913514 | 2.04055 | -0.47885 | 0.672306 | -0.0518245 |
| CTC | 0.847362 | 2.03043 | 0.548342 | 0.55447 | 0.0593455 |
| AGT | 0.666666 | 2.32204 | 1.63807 | 0.442167 | 0.177283 |
| CGT | 0.983781 | 1.8522 | -2.31964 | 0.875644 | -0.251048 |
| GCC | 0.91735 | 2.01123 | -0.674459 | 0.689912 | -0.0729947 |
| CTC | 0.761539 | 2.08614 | 1.27782 | 0.471954 | 0.138295 |
| ATC | 0.756492 | 2.10574 | -1.88623 | 0.82071 | -0.204141 |
| TTC | 0.812806 | 2.00388 | -0.429933 | 0.688247 | -0.0465304 |
| AAT | 0.527396 | 2.38415 | -2.45067 | 0.817206 | -0.265229 |
| ATC | 0.77969 | 1.96889 | -0.349656 | 0.667711 | -0.0378422 |
| TTG | 0.871641 | 2.10883 | -1.19322 | 0.745531 | -0.129139 |
| TTC | 0.964533 | 1.89104 | 0.125074 | 0.592782 | 0.0135364 |
| AAC | 0.86428 | 2.07449 | -2.26628 | 0.855083 | -0.245273 |
| AGT | 0.813049 | 2.12136 | 1.67978 | 0.414022 | 0.181798 |
| GCC | 0.565472 | 2.41276 | -2.10018 | 0.827655 | -0.227296 |
| AAT | 0.930828 | 2.05557 | -0.608088 | 0.683284 | -0.0658115 |
| GTG | 0.704415 | 2.24775 | -0.297315 | 0.667942 | -0.0321776 |
| CAA | 0.707827 | 2.24365 | 1.55581 | 0.442373 | 0.168381 |
| GTG | 0.600711 | 2.07967 | 4.90052 | 0.16379 | 0.530369 |
| TGG | 0.931543 | 2.06846 | 1.72064 | 0.355232 | 0.18622 |
| GAT | 0.93811 | 2.05206 | 2.93919 | 0.225144 | 0.3181 |
| CAG | 0.976694 | 1.99653 | -1.40334 | 0.762561 | -0.151879 |
| GGC | 0.728668 | 2.00146 | 0.133063 | 0.602786 | 0.0144011 |
| CCT | 0.703238 | 2.28774 | -0.697147 | 0.713933 | -0.0754502 |
| TTT | 0.954248 | 2.01316 | -2.58943 | 0.875632 | -0.280246 |
| CGC | 0.616408 | 2.33745 | 4.88109 | 0.132056 | 0.528266 |
| CCT | 0.752321 | 2.19845 | -0.750536 | 0.704652 | -0.0812283 |
| CAC | 0.538658 | 2.27363 | -0.73132 | 0.72696 | -0.0791486 |
| CGC | 0.858961 | 2.10932 | -2.58936 | 0.858316 | -0.280239 |
| CTC | 0.683007 | 2.05798 | 1.92772 | 0.403855 | 0.208631 |
| CTC | 0.824259 | 2.00495 | 2.20401 | 0.334955 | 0.238533 |

| GCA/TGC Motif | | | | | |
| --- | --- | --- | --- | --- | --- |
| Triplet | Syn sites (S) | Nonsyn sites (N) | dN-dS | Prob. (positive selection) | Normalized dN-dS |
| TGA | 0.964105 | 1.9946 | -0.696999 | 0.696479 | -0.0753993 |
| CGG | 0.975758 | 2.02424 | -1.20895 | 0.760088 | -0.13078 |
| CTT | 0.956179 | 1.99022 | -0.734321 | 0.70043 | -0.0794367 |
| CGA | 0.524856 | 1.94715 | -3.36356 | 0.867423 | -0.363861 |
| ACT | 0.778618 | 1.84346 | -0.797655 | 0.73168 | -0.086288 |
| CTG | 0.965429 | 1.92443 | 0.0138471 | 0.629397 | 0.00149794 |
| TTT | 0.598079 | 2.22956 | -1.75438 | 0.804831 | -0.189784 |
| T-C | 0.919839 | 1.95805 | 0.758547 | 0.519949 | 0.0820575 |
| TGT | 0.914656 | 1.8478 | -1.13706 | 0.764196 | -0.123004 |
| TGC | 0.870237 | 2.06078 | -1.86354 | 0.840912 | -0.201592 |
| AAA | 1.00098 | 1.98028 | -1.46025 | 0.817387 | -0.157965 |
| CTG | 1.01393 | 1.9579 | -0.369786 | 0.691133 | -0.0400024 |
| GGT | 0.889207 | 2.08062 | 1.26911 | 0.451958 | 0.137289 |
| TTT | 0.791561 | 2.03542 | -0.660827 | 0.68159 | -0.0714863 |
| TGG | 0.915063 | 1.84618 | 3.5097 | 0.139231 | 0.379669 |
| GTG | 0.923466 | 2.04116 | -0.218732 | 0.648599 | -0.0236618 |
| TTT | 0.645228 | 2.11009 | -1.39617 | 0.785208 | -0.151033 |
| TAA | 0.579224 | 2.39066 | -2.30456 | 0.848339 | -0.249301 |
| GTG | 1.05234 | 1.93305 | -0.0954722 | 0.632961 | -0.0103279 |
| TTG | 0.98962 | 1.83866 | -0.39089 | 0.659854 | -0.0422854 |
| GTC | 0.874017 | 2.0182 | 0.531494 | 0.563118 | 0.0574956 |
| GTG | 0.700909 | 1.96242 | 2.02161 | 0.396112 | 0.218692 |
| GTG | 0.919481 | 2.03356 | -0.92235 | 0.705755 | -0.0997772 |
| ACG | 1.05139 | 1.92144 | 1.31019 | 0.415418 | 0.141733 |
| AGA | 1.04619 | 1.91469 | -0.256171 | 0.699221 | -0.0277119 |
| GCA | 1.03101 | 1.96899 | 0.891654 | 0.488462 | 0.0964566 |
| AAG | 0.929835 | 2.04692 | 0.191718 | 0.584647 | 0.0207395 |
| AGA | 1.04672 | 1.94018 | -0.238711 | 0.671239 | -0.0258231 |
| TGG | 1,0 | 1.98193 | 1.02735 | 0.463264 | 0.111136 |
| GAA | 0.916272 | 2.04427 | 0.714288 | 0.52262 | 0.0772697 |
| TGG | 0.85646 | 2.09325 | -0.246696 | 0.636585 | -0.0266869 |
| TTT | 0.963607 | 2.03639 | -1.76993 | 0.829636 | -0.191466 |
| TGT | 0.97168 | 1.92199 | -1.4884 | 0.776547 | -0.161011 |
| CTG | 0.537339 | 2.00393 | 5.23092 | 0.150389 | 0.565866 |
| TGA | 0.96722 | 1.9158 | -1.24627 | 0.761683 | -0.134817 |
| CGC | 0.95481 | 2.01595 | 2.8842 | 0.20284 | 0.312004 |
| GAC | 1.00688 | 1.87989 | 0.400764 | 0.556512 | 0.0433535 |
| GCC | 0.635713 | 2.31333 | -1.6752 | 0.781595 | -0.181219 |
| TGA | 0.505971 | 2.2175 | -3.39599 | 0.898789 | -0.367369 |
| GTT | 0.708556 | 2.2249 | -0.251788 | 0.660457 | -0.0272377 |
| GTT | 0.886818 | 2.0313 | -0.715176 | 0.710051 | -0.0773657 |
| GAT | 0.929214 | 2.0636 | 0.883099 | 0.498664 | 0.0955312 |
| GAT | 0.927989 | 1.98006 | -1.34771 | 0.792787 | -0.145791 |
| CCG | 0.946245 | 2.04709 | 0.373589 | 0.566762 | 0.0404138 |
| AGA | 0.933718 | 2.0322 | 1.12892 | 0.465065 | 0.122123 |
| TTG | 1.17376 | 1.77383 | 0.52575 | 0.533089 | 0.0568742 |
| TGG | 0.710647 | 1.91497 | 4.25732 | 0.121616 | 0.460545 |
| AGG | 0.65716 | 2.2822 | 1.56934 | 0.452431 | 0.169767 |
| TTT | 0.849345 | 1.95905 | 4.28112 | 0.122747 | 0.463119 |
| GTT | 0.858695 | 1.98593 | -2.95901 | 0.904002 | -0.320097 |
| TTC | 0.840499 | 2.09835 | 3.81582 | 0.152434 | 0.412784 |
| TTG | 0.907337 | 2.06006 | 0.445719 | 0.565308 | 0.0482166 |
| CTG | 0.904673 | 2.09533 | -0.113891 | 0.622613 | -0.0123204 |
| CTG | 0.836133 | 1.97673 | 1.4095 | 0.440132 | 0.152475 |
| TGA | 0.867966 | 2.09405 | -0.844905 | 0.707967 | -0.0913994 |
| AGT | 0.959524 | 2.00338 | 0.595189 | 0.539524 | 0.0643858 |
| TGC | 1.03413 | 1.94442 | 1.02805 | 0.467732 | 0.111211 |
| GTG | 0.927491 | 2.03728 | 0.82515 | 0.506937 | 0.0892623 |
| GGC | 1.01648 | 1.86363 | 2.9511 | 0.213788 | 0.319242 |
| CTA | 0.619569 | 2.32578 | 4.47824 | 0.1214 | 0.484443 |
| CGG | 0.854097 | 2.07833 | 0.954245 | 0.497531 | 0.103228 |
| GTA | 1.0445 | 1.86508 | -2.46023 | 0.855208 | -0.26614 |
| GGT | 0.92804 | 1.90459 | -0.109778 | 0.64778 | -0.0118755 |
| GCG | 0.456573 | 2.05027 | 3.4234 | 0.337702 | 0.370334 |
| TGG | 0.609488 | 2.32016 | -1.04676 | 0.725875 | -0.113235 |
| ATT | 0.966533 | 1.99942 | -1.70644 | 0.814532 | -0.184598 |
| GAA | 0.697496 | 2.28822 | 0.380888 | 0.596604 | 0.0412034 |
| AAT | 0.959939 | 2.04006 | -1.28721 | 0.790774 | -0.139247 |
| CCA | 1.15799 | 1.74198 | 1.34791 | 0.423878 | 0.145813 |
| CAG | 0.955497 | 1.98314 | -0.152298 | 0.654781 | -0.0164752 |
| TTG | 0.80843 | 1.92657 | 0.988142 | 0.500227 | 0.106894 |
| TCA | 1.01052 | 1.95621 | -1.3368 | 0.779021 | -0.144612 |
| TTT | 0.527022 | 2.22695 | 4.35286 | 0.159572 | 0.47088 |
| GGG | 0.609576 | 2.30337 | 1.32563 | 0.488665 | 0.143403 |
| CAG | 0.877787 | 2.11017 | -0.342059 | 0.649566 | -0.037003 |
| TTT | 0.622635 | 2.25852 | 2.40493 | 0.368859 | 0.260159 |
| GAT | 0.666569 | 2.27578 | -1.69776 | 0.78998 | -0.183659 |
| CGA | 0.967111 | 2.0163 | -1.01754 | 0.721065 | -0.110074 |
| AAT | 0.916186 | 2.07558 | -2.87149 | 0.914505 | -0.31063 |
| CGC | 0.936391 | 1.99547 | 1.26265 | 0.446669 | 0.13659 |
| AGC | 0.957435 | 2.01966 | -1.97683 | 0.872543 | -0.213848 |
| AAG | 0.596464 | 1.99559 | -1.52192 | 0.820359 | -0.164637 |
| GCT | 0.60313 | 1.94089 | -0.224675 | 0.71043 | -0.0243047 |
| GTA | 0.571316 | 2.01791 | -1.28653 | 0.790337 | -0.139173 |
| GCA | 0.663421 | 1.90317 | -4.84051 | 0.954666 | -0.523633 |
| GTC | 0.63756 | 1.98423 | -0.701934 | 0.708974 | -0.0759333 |
| GTG | 0.875897 | 2.10261 | 0.601361 | 0.540245 | 0.0650535 |
| GGC | 0.565422 | 2.37087 | -0.130901 | 0.647765 | -0.0141605 |
| CAG | 0.693022 | 2.29678 | -0.356401 | 0.672688 | -0.0385544 |
| GTT | 0.573238 | 2.38855 | -1.46546 | 0.811441 | -0.158529 |
| GTT | 0.560504 | 2.34006 | -0.651587 | 0.721229 | -0.0704868 |
