# Supplementary material for: In silico comparative analysis of SSR markers in plants
Source: BMC Plant Biol. 2011 Jan 19;11:15. doi: 10.1186/1471-2229-11-15 (PMC3037304; doi:10.1186/1471-2229-11-15)
Supplement: Additional file 8 — Eletronical PCR results table. [file 1471-2229-11-15-S8.DOC]

**Additional file 8.** Eletronical PCR results

| *Chlamydomonas* EST-SSR | | | | |
| --- | --- | --- | --- | --- |
| Total rimers | Species | | Positives Primers | Tranferability (%) |
| 319 | *Mesostigma viride* | | 1 | 0,3 |
|  | *Marchantia polymorpha* | | 1 | 0,3 |
|  | *Syntrichia ruralis* | | 9 | 2,8 |
|  | *Physcomitrella pates* | | 7 | 2,2 |
|  | *Selaginella ssp* | | 2 | 0,6 |
|  | *Adiantum capillus-veneris* | | 86 | 26,9 |
|  | *Gnetum gnemon* | | 3 | 0,94 |
|  | *Pinus taeda* | | 4 | 1,2 |
|  | *Oryza sativa* | | 8 | 2,5 |
|  | *Arabidopsis thaliana* | | 29 | 9 |
| *Arabidopsis* EST-SSR | | | | |
| Total Primers Arabidopsis | | Species | Primers Positivos | % |
| 1250 | | *Mesostigma viride* | 1 | 0,08 |
|  | | *Marchantia polymorpha* | 1 | 0,08 |
|  | | *Syntrichia ruralis* | 4 | 0,32 |
|  | | *Physcomitrella pates* | 4 | 0,32 |
|  | | *Selaginella ssp* | 13 | 1,04 |
|  | | *Adiantum capillus-veneris* | 6 | 0,48 |
|  | | *Gnetum gnemon* | 9 | 0,72 |
|  | | *Pinus taeda* | 5 | 0,4 |
|  | | *Oryza sativa* | 15 | 1,2 |
|  | | *Arabidopsis thaliana* | 24 | 1,92 |
